# Discovery and Validation of SIRT2 Inhibitors Based on Tenovin-6: Use of a ^1^H-NMR Method to Assess Deacetylase Activity

**DOI:** 10.3390/molecules171012206

**Published:** 2012-10-18

**Authors:** Lisa Pirrie, Anna R. McCarthy, Louise L. Major, Vaida Morkūnaitė, Asta Zubrienė, Daumantas Matulis, Sonia Lain, Tomas Lebl, Nicholas J. Westwood

**Affiliations:** 1 School of Chemistry and Biomedical Sciences Research Complex, University of St Andrews and EaStCHEM, North Haugh, St Andrews, Fife KY16 9ST, UK; 2 Department of Biothermodynamics and Drug Design, Institute of Biotechnology, Vilnius University, Graiciuno 8LT-02241, Vilnius, Lithuania; 3 Department of Microbiology, Tumor and Cell Biology, Karolinska Institutet, Stockholm SE-17177, Sweden; 4 Centre for Oncology & Molecular Medicine, University of Dundee, Ninewells Hospital & Medical School, Dundee DD1 9SY, UK

**Keywords:** sirtuin, chemical tool, deacetylase assay, neurodegenerative diseases

## Abstract

The search for potent and selective sirtuin inhibitors continues as chemical tools of this type are of use in helping to assign the function of this interesting class of deacetylases. Here we describe SAR studies starting from the unselective sirtuin inhibitor tenovin-6. These studies identify a sub-micromolar inhibitor that has increased selectivity for SIRT2 over SIRT1 compared to tenovin-6. In addition, a ^1^H-NMR-based method is developed and used to validate further this class of sirtuin inhibitors. A thermal shift analysis of SIRT2 in the presence of tenovin-6, **-**43, a control tenovin and the known SIRT2 inhibitor AGK2 is also presented.

## 1. Introduction

The identification and optimisation of sirtuin inhibitors has received considerable attention in recent years including our own contributions to the optimisation of cambinol and the discovery of the tenovins [1,2,3,4,5,6,7,8,9]. The sirtuins, of which there are seven human isoforms SIRT1-SIRT7, belong to the class III family of histone deacetylases (HDACs) [10].The major role of these enzymes is as NAD^+^-dependent deacetylases of histone and non-histone substrates although some members of this protein family possess ADP ribosylase activity, and more recently SIRT5 has been shown to possess NAD^+^ dependant demalonylase and desuccinylase activities [11]. SIRT1 has a broad set of substrates including the important tumour suppressor p53 and inhibition of SIRT1 has therefore been linked to anti-cancer therapy [12]. A very recent report describes the use of tenovin-6 in combination with imatinib (a BCR-ABL kinase inhibitor) in a mouse model of chronic myeloid leukemia (CML). Treatment with both compounds has been shown to result in significant loss of CML stem cells, a result that cannot be achieved by the use of imatinib alone since CML stem cells do not require BCR-ABL to replicate [13,14,15]. The authors propose that this enhanced killing of the stem cells results from the inhibition of SIRT1 by tenovin-6. Tenovin-6 was also shown to be effective in a cell culture model for CML acquired resistance where treatment of cells with tenovin-6 blocked the acquisition of BCR-ABL mutations. When used in combination with imatinib high levels of apoptosis were observed [13,14,15]. Inhibition of SIRT2, whose known substrates include tubulin and histones H3 and H4, has been studied in the context of neurodegenerative diseases [16,17]. For example AGK2, a SIRT2 selective inhibitor (IC_50_ = 3.5 µM), has already been used to provide novel insights into aspects of both Parkinson’s and Huntington’s disease [18,19]. Whilst the use of AGK2 provides an important approach to studying SIRT2 function, the development of additional chemical tools may also prove beneficial. Here we report on the discovery of a sub-micromolar inhibitor of SIRT2 that has been validated using a novel ^1^H-NMR-based method.

## 2. Results and Discussion

### 2.1. Chemistry

**Scheme 1:**
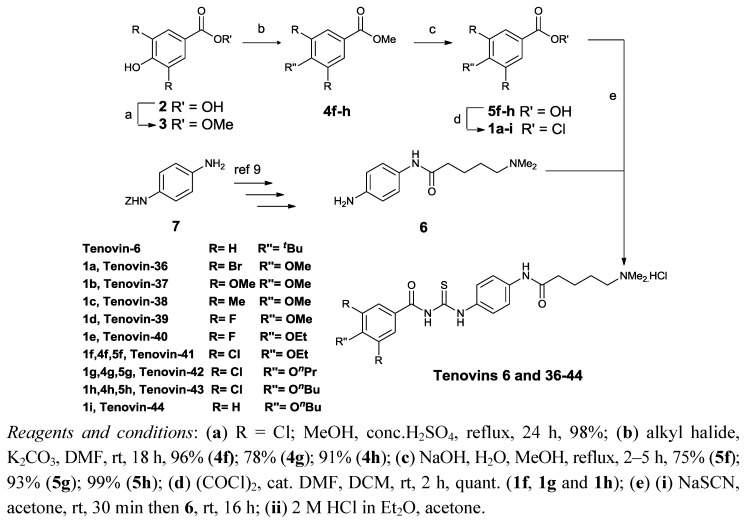
Synthesis of tenovin analogues.

Initial studies generated structure activity relationships for tenovin-6 (Scheme 1 and Table 1). Previously we have shown that modification of the substituents in the *N*-benzoyl ring of tenovin-6 led to more active analogues and it was therefore decided to prepare additional analogues substituted in this region to tune selectivity towards SIRT2 over SIRT1 [9]. In brief, the synthesis of the novel tenovin-6 analogues followed our published route (Scheme 1) [9]. Whilst several of the acid chlorides **1** were available (compounds **1a**–**e**,**i**), the acid chlorides **1f**–**h** (see Scheme 1 for substituents) required for the synthesis of tenovins 41–43 were synthesised in four steps from the corresponding 4-hydroxycarboxylic acid **2** (R = Cl) as follows: esterification of the carboxylic acid **2** using standard conditions was followed by O-alkylation of the corresponding ester **3** (R = Cl) using the required alkyl halides to give **4f**–**h**. Hydrolysis of the ester functionality in **4f**–**h** followed by reaction of the resulting acids **5f**–**h** with oxalyl chloride afforded the required acid chlorides **1f**–**h** in high overall yields. The acid chlorides **1a**–**i** were then coupled with amine 6, synthesised in four steps from *p*-phenylenediamine (**7**), to give the required tenovin-6 analogues [9].

**Table 1:** Activity of novel tenovin analogues against SIRT1 and SIRT2.

Tenovin	R	R′′	SIRT1	IC_50_	SIRT2	IC_50_	Selectivity Factor
			% at 60 µm ^a^		% at 60 μM ^a^		
6	H	*^t^*Bu	73.9 ± 1	21^b^	94.9 ± 1	10 ^b^	2.1
36	Br	OMe	66.2 ± 2	51.6 ± 2	82.7 ± 1	16.0 ± 3	3.2
37	OMe	OMe	12.5 ± 1	n.d.	18.1 ± 1	n.d.	n.d.
38	Me	OMe	34.2 ± 3	65.4 ± 1	54.7 ± 1	44.9 ± 1	1.5
39	F	OMe	79.5 ± 4	47.2 ± 1	81.2 ± 5	18.0 ± 3	2.6
40	F	OEt	72.0 ± 4	35.3 ± 3	89.1 ± 1	17.5 ± 2	2.0
41	Cl	OEt	79.4 ± 1	28.5 ± 1	81.5 ± 2	12.9 ± 1	2.2
42	Cl	O^n^Pr	81.0 ± 3	23.5 ± 2	96.1 ± 1	4.7 ± 3	5.0
43	Cl	O^n^Bu	89.2 ± 2	21.5 ± 1	99.6 ± 3	0.8 ± 0.4	26.9
44	H	O^n^Bu	87.7 ± 1	36.5 ± 5	88.2 ± 2	7.2 ± 1	5.1

^a^ % inhibition measured at 60 μM concentration of inhibitor ± SE (standard error, n = 2); ^b^ see reference 8; n.d. not determined.

### 2.2. *In Vitro* Inhibition of SIRT1 and SIRT2

**Figure 1:**
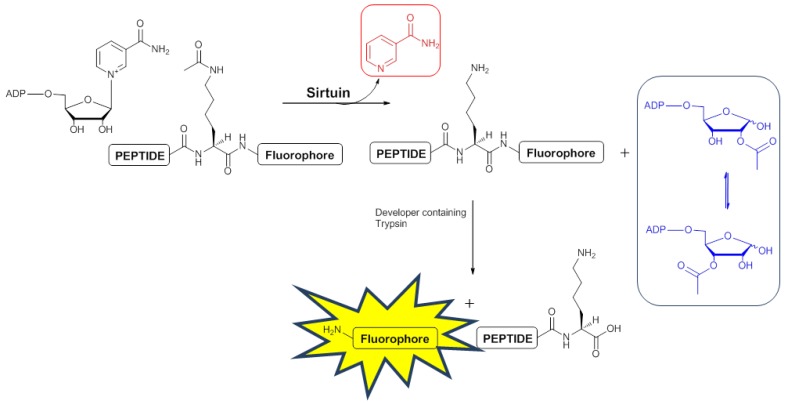
Commercially available sirtuin assay uses a fluorescently labelled peptide substrate containing an *N*-acetylated lysine residue [20]. Removal of the *N*-acetyl group is coupled with conversion of NAD^+^ to nicotinamide (in red), 2′-*O*-acetyl ADP-ribose (in blue) and the deacetylated substrate. Subsequent reaction with trypsin releases the quenched fluorophore.

The assessment of a compound’s ability to inhibit SIRT2 function *in vitro* is frequently carried out within the sirtuin community using a commercially available assay [20]. This assay, which relies on the deacetylation of a fluorescently labelled acetylated peptide substrate (Figure 1), was initially used here (Table 1). 

The 3,5-dibromo-4-methoxy-substituted analogue, tenovin-36 showed reduced activity against SIRT2 compared with tenovin-6 but was even less active against SIRT1 (c.f. Table 1, entries 1 and 2). It was decided to explore this substitution pattern further. Replacement of the 3,5-halogen atoms in tenovin-36 by electron-donating substituents, OMe or Me (tenovins-37 and -38) led to a detrimental effect on both potency and selectivity. Returning to 3,5-dihalo-substituted analogues, tenovin-39 showed a similar activity profile to tenovin-36 (c.f. entries 2 and 5). Increasing the size of the alkyl chain in the 4-alkoxy-substituent from OMe in tenovin-39 to OEt in tenovin-40 led to little improvement. On moving back to chloro-substituents in the OEt series, the activity and selectivity was retained and it was demonstrated that further increases in the alkoxy chain length led to a significant increase in activity towards SIRT2, while the SIRT1 activity was almost unchanged (tenovins 41-43, c.f. entries 7–9). Tenovin-43 is, to the best of our knowledge, one of the most potent SIRT2 inhibitors reported to date [21,22]. Removal of the chloro substituents present in tenovin-43 to give tenovin-44 had a detrimental effect on activity.

### 2.3. Development of a ^1^H-NMR Assay for Deacetylase Activity

With the increase in the use of high-throughput screening as a starting point for chemical tool and drug discovery projects, attention has turned to methods by which false positives can arise from screening campaigns. For example, stabilisers of the luciferase enzyme and compounds that quench the fluorescence in neuraminidase assays have recently been shown to produce misleading results in HT assays [23,24]. In addition there has been some challenges reported with the commercial sirtuin assay used here [25]. With this in mind, we decided to generate an alternative method to validate our hits using ^1^H-NMR methods. This approach is appealing because it would use a non-fluorescently labelled substrate to enable the direct monitoring of the deacetylation of the substrate to give the product peptide. It would also allow the production of additional reaction products such as nicotinamide or 2'/3'-O-acetyl ADP-ribose to be monitored giving multiple readouts. 

#### 2.3.1. Deacetylation of a Histone H4 Peptide by SIRT2

**Figure 2:**
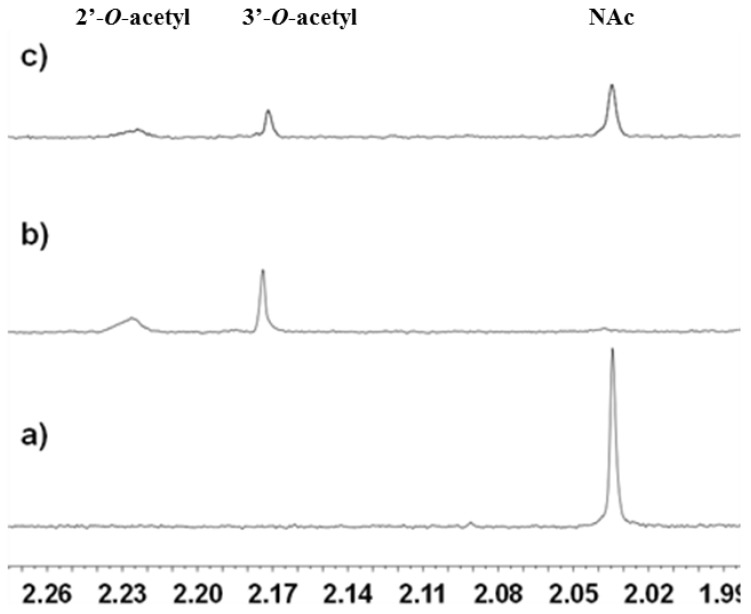
1D ^1^H-NMR with double solvent suppression (for both H_2_O and Tris buffer) recorded at 37 °C. Sample contained 1 mM NAD^+^, 200 µM peptide, 10 µM SIRT2 in buffer (pH 8). (**a**) Before addition of enzyme. (**b**) After addition of enzyme and 15 mins. incubation. (**c**) Sample containing 200 μM tenovin-6 after incubation with enzyme for 15 min. Throughout this work, the H4 substrate was used at a final concentration of 200 μM as this enabled monitoring using a reasonable number of scans.

As described above, histone H4 is a known substrate of SIRT2 and it was therefore decided to use the 11 amino acid-containing peptide GLGKGGAK(Ac)RHR based on H4 as the SIRT2 substrate [16]. A C-terminal His-tagged version of the human sirt2 gene corresponding to residues 50 to 356 was cloned into a pET32a vector and the protein overexpressed using BL21(DE3) cells. Standard purification methods were used to access untagged SIRT2 in large quantities. Encouragingly, SIRT2-mediated deacetylation of the H4 substrate was observed by ^1^H-NMR (Figure 2). When the H4 substrate was characterised by ^1^H-NMR a peak at 2.03 ppm assigned to the acetyl methyl group was observed (Figure 2a). Band-selective ^1^H, ^13^C-HMBC experiments confirmed the assignment was correct (See Figures S2 and S3).

**Figure 3:**
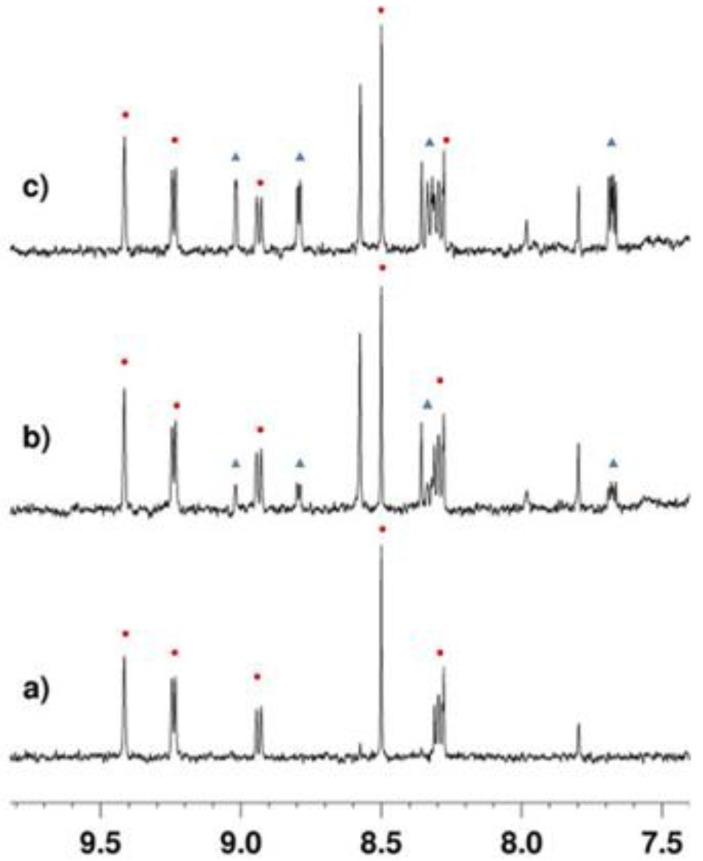
1D ^1^H-NMR with double solvent suppression (H_2_O and Tris buffer) recorded at 37 °C. (**a**) before addition of enzyme; (**b**) after incubation with SIRT2 for 20 mins at 37 °C. (**c**) after addition of authentic nicotinamide. Circles: signals from NAD^+^; Triangles signals from nicotinamide.

Reaction of the H4 substrate with SIRT2 for 15 min at 37 °C followed by reanalysis led to the spectrum shown in Figure 2b in which the signal assigned to the lysine *N*-acetyl group in the substrate had disappeared (c.f. Figure 2a). The new signals at 2.17 ppm and 2.22 ppm (Figure 2b,c) were assigned as the acetyl groups in the *O*-acetylated ADP-ribose products formed from NAD^+^ (Figure 1) as determined by doping of authentic material into the reaction mixture (Figures S4 and S5). Doping experiments using an authentic sample of the deacetylated peptide (Figure S6) also confirmed that the H4 substrate was deacetylated by SIRT2. Finally, the observation of a new set of signals in the aromatic region of the spectrum were explained by the formation of nicotinamide from NAD^+^ (blue triangles in Figure 3b), consistent with the expected reaction. Doping of the final reaction mixture with authentic nicotinamide confirmed that it was formed in the SIRT2-catalysed reaction (Figure 3c). 

#### 2.3.2. Inhibition of the Deacetylase Reaction of SIRT2 by the Tenovins

**Figure 4:**
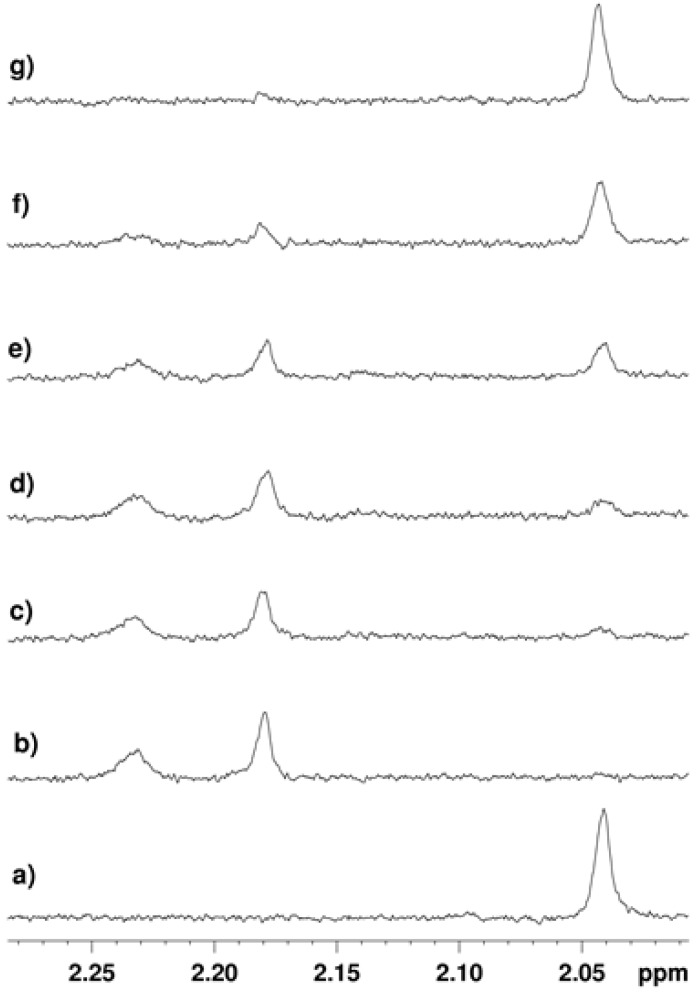
^1^H-NMR spectrum obtained: (**a**) before addition of SIRT2; (**b**) 8 min after addition of SIRT2; the *N*-acetyl signal had disappeared consistent with substrate turnover by SIRT2. (**c**–**g**) 8 min after addition of enzyme with **tenovin-6** at a final concentration of: (**c**) 25 µM; (**d**) 50 µM. (**e**) 100 µM; (**f**) 200 µM; (**g**) 500 µM.

Having shown that deacetylation of the H4 substrate could be monitored by ^1^H-NMR, it was decided to assess whether tenovin-6 (Scheme 1) could inhibit the reaction. When the free base of tenovin-6 was used (final concentration 200 μM) a significant reduction in the amount of substrate that was deacetylated by SIRT2 in 15 min was observed (c.f. Figure 2b,c) consistent with SIRT2 inhibition by tenovin-6. More detailed studies across a 0–500 µM final concentration range of tenovin-6 showed that the degree of inhibition observed was dose-dependent (Figure 4). The degree of inhibition was quantified by integration of the *N*-acetyl methyl group of the starting peptide with respect to the satellite peak of the TRIS buffer, the concentration of which was constant for all samples (Table S1). From this data an IC_50_ of 139.2 ± 9.5 µM was calculated for tenovin-6.

**Figure 5:**
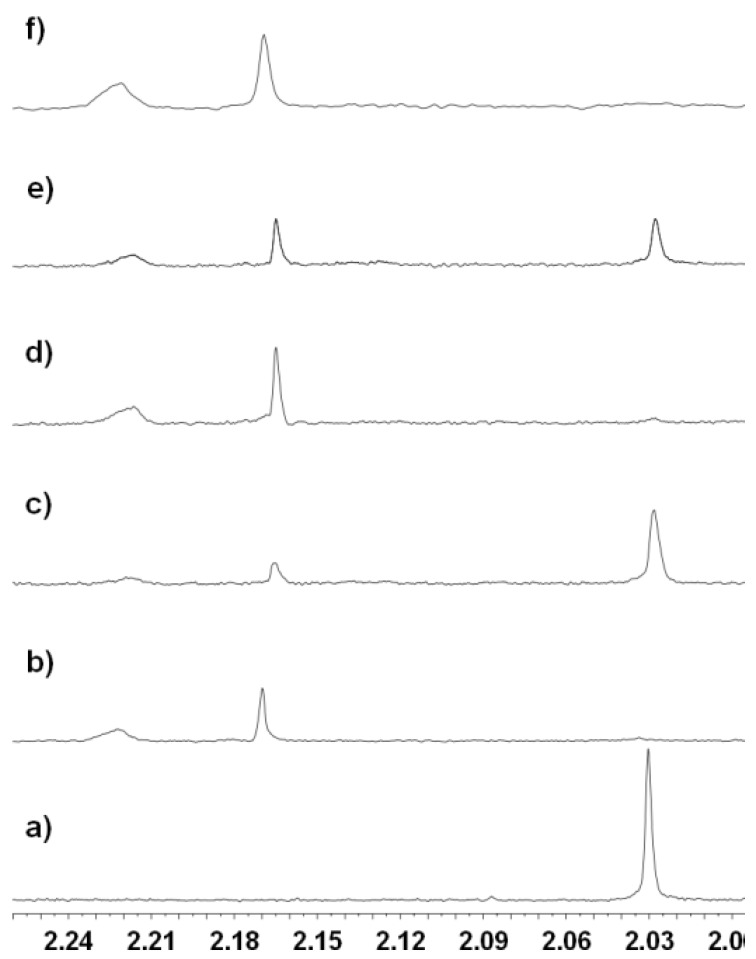
The ^1^H-NMR spectrum obtained: (**a**) before addition of enzyme; (**b**–**f**) 20 min after addition of: (**b**) SIRT2; (**c**) SIRT2 and tenovin-43 (200 µM); (**d**) SIRT2 with tenovin-6 (25 µM); (**e**) SIRT2 with tenovin-43 (25 µM); (**f**) SIRT2 with AGK2 (25 µM).

The effect of the new SIRT2 selective inhibitor, tenovin-43 was then assessed. Inhibition of SIRT2 by tenovin-43 was observed, after a 20 min incubation period (c.f. the 8 min incubation period used to generate the data in Figure 4). A large signal corresponding to the *N*-acetyl in the H4 substrate was observed when tenovin-43 was present at a final concentration of 200 μM [compare Figure 5a (no enzyme) 5b (+SIRT2) and 5c (+SIRT2 and tenovin-43)]. Inhibition was also observed for tenovin-43 at a lower concentration of 25 µM with a signal corresponding to the presence of the *N*-acetylated peptide still present after the 20 min incubation (Figure 5e). However, the corresponding signal was not observable upon incubation with tenovin-6 at 25 µM under otherwise identical conditions (Figure 5d). On incubation with AGK2, the current state of the art SIRT2 inhibitor, no inhibition of SIRT2 at a final concentration of AGK2 of 25 μM was observed (Figure 5f) [19]. Due to the low solubility of AGK2 it was not possible to carry out this reaction at higher final concentrations of AGK2. Although direct comparison of the results obtained in the ^1^H-NMR assay with the commercially available assay is difficult, due to differences in the structure and concentration of the substrate, it is clear that analogous trends for inhibitor potency are seen in the two assays.

### 2.4. Thermal Shift Analysis of SIRT2 in the Presence of the Tenovins and AGK2

**Figure 6:**
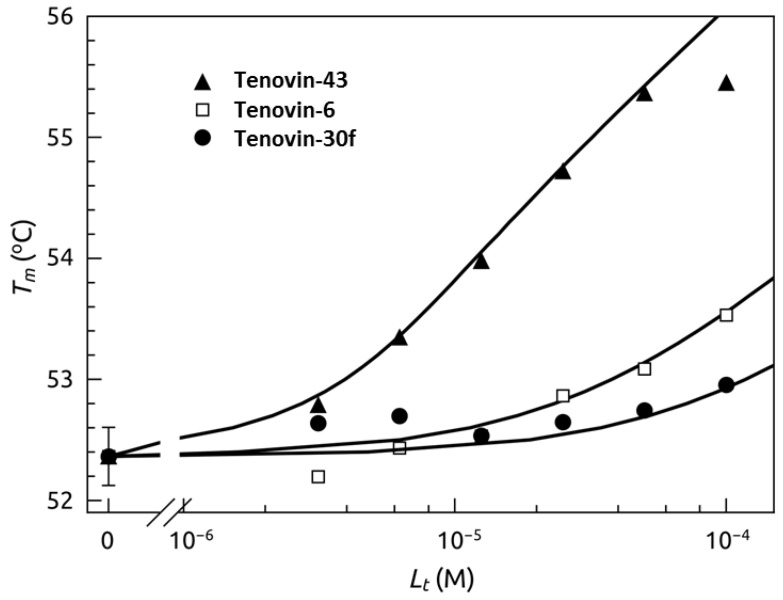
Ligand dosing curves showing the T_m_ shift dependence of SIRT2 on ligand concentration. Datapoints are experimental data obtained from curves as in Figure S6 while the lines are simulated according to the model as previously explained [26,27,28,29,30,31,32]. The dissociation constants at 37 °C, for the three ligands were: 0.67 µM for tenovin-43, 15 µM for tenovin-6 and 50 µM for tenovin-30f [9].

The binding of tenovins (tenovin-6, tenovin-43, and a previously reported inactive analogue tenovin-30f) to SIRT2 was also determined by a fluorescent thermal shift assay (Figure 6 and Figure S7) [9]. The dissociation constants at 37 °C, for the three ligands were: 0.67 µM for tenovin-43, 15 µM for tenovin-6, and 50 µM for the previously reported inactive tenovin-30f [9]. The calculated K_d_ values as determined by thermal shift correlate well with the previously obtained data using both the commercially available assay kit and the NMR method, where the most potent analogue tenovin-43 was shown to have the lowest dissociation constant. AGK2 was also assessed by thermal shift and was determined to have a K_d_ of >200 µM (Figures S8 and S9). 

## 3. Experimental

### 3.1. SIRT2 Expression and Purification

SIRT2 cDNA (residues 50-356) was expressed from Ncol and Xhol restriction sites of the pET32a vector (Novagen, Merck Millipore, Billerica, MA, USA) with an N-terminal His tag. Protein was overexpressed in BL21(DE3) pLysS bacterial cells (Sigma, Dorset, UK). Competent bacteria were transformed with the SIRT2 plasmid and starter cultures (10 mL) grown overnight at 37 °C in LB medium with ampicillin (100 mg/L) and chloramphenicol (100 mg/L). The overnight starter culture was added to 1 L of LB media containing ampicillin and chloramphenicol which was grown at 37 °C with 200 rpm shaking, until the OD_600_ reached 0.7–0.8. Cultures were cooled on ice before the addition of IPTG (0.1 mM) and Zn(OAc)_2_ (40 mM) and the culture grown at 18 °C overnight. The cells were harvested by centrifugation at 7500 rpm for 35 min before storing the resulting pellets at −20 °C. SIRT2 containing pellets were resuspended in lysis buffer (20 mM Tris pH 8, 200 mM NaCl, 10 mM imidazole, 2 mM β-mercaptoethanol, Roche EDTA-free mini protease inhibitor cocktail) and incubated on ice for 30 min. The suspension was sonicated for 10 × 1 min. The soluble fraction was collected after centrifugation at 15,000 rpm for 30 min. The resulting supernatant was filtered (0.45 µm), and loaded onto a pre-equilibrated 5mL HisTrap HP column (GE Healthcare, UK). The tagged protein eluted at 100 mM imidazole concentration. The fractions containing the enzyme were combined and dialysed in buffer (50 mM Tris pH 7.5, 100 mM NaCl, 5 mM imidazole, 2 mM β-mercaptoethanol) for 2 h. The protein was placed in fresh dialysis buffer and biotinylated thrombin added before leaving to cleave overnight at 4 °C. The enzyme was transferred into a tube where streptavidin agarose was added and the mixture gently mixed on a rocking table for 2 h at 4 °C. The suspension was centrifuged at 3,000 rpm for 10 min and the supernatant containing the purified enzyme decanted. The cleaved SIRT2 was further purified by Ni affinity chromatography as before, with cleaved protein eluting at 5 mM imidazole, followed by gel filtration chromatography on S-200 sephacryl column. The protein was concentrated by centrifugation using a spin concentrator. SIRT2 was concentrated to >12 mg/mL to ensure minimal amount of water in the NMR experiments.

### 3.2. SIRT1 and SIRT2 Inhibition Assay

All compounds were tested for inhibition of SIRT1 and SIRT2 using human recombinant enzymes; SirT1 available in the Fluor de Lys^®^ fluorescence-based assay kit (Enzo Life Sciences, AK555, Exeter, UK) and SirT2 expressed and purified in house. All other required reagents were provided in the kit which was stored at −78 °C. Before use, small aliquots of each enzyme (2–3 μL) were prepared, snap frozen in liquid nitrogen and stored at −78 °C. Fresh dilutions of compounds were prepared in DMSO and further diluted in assay buffer. NAD^+^ (12.5 μL at 4 mM) and Fluor de Lys^®^ SIRT1 or SIRT2 (12.5 μL at 100 μM) in assay buffer were added to a white 96 well plate, followed by compound (10 μL) and lastly enzyme (15 μL, 0.07 U/μL for kit SIRT1 and 0.3 U/μL for in-house SIRT2). After incubation for 1 h at 37 °C a developer solution (50 μL) was added to each reaction. The developer solution contained 38 μL buffer, 10 μL developer and 2 μL nicotinamide per reaction. The plate was then incubated for 45 min at rt and then read using a Spectra Max Gemini fluorimeter with an excitation wavelength of 355 nm and an emission wavelength of 460 nm. SigmaPlot software was used to generate fit curves for raw plots and the equation for each fit curve was used to calculate IC_50_ data.

### 3.3. ^1^H-NMR Experiments

To a D_2_O buffer containing 50 mM Tris (pH 8), 150 mM NaCl, 2 mM KCl and 1 mM MgCl_2_ was added NAD^+^ (1 mM), peptide (200 μM) and inhibitor (varying concentration). The inhibitor was dissolved in *d_6_*-DMSO before addition to the buffer ensuring that the final NMR sample contained only 0.5% DMSO for each experiment. The initial data was collected before addition of in house SIRT2 (10 μM) directly to the NMR tube. After the incubation was complete, the NMR tube was shaken and replaced in the spectrometer for data collection. 1D ^1^H-NMR with double solvent suppression (for H_2_O and TRIS) were recorded on a 500 MHz Bruker spectrometer at 37 °C.

### 3.4. Thermal Shift Experiments

The thermal shift assay was performed using Corbett Rotor-Gene 6000 (QIAGEN Rotor-Gene Q, Qiagen, Sydney, Australia) spectrofluorimeter. The prepared protein concentration was usually 5 µM and the ligand concentrations 50 µM. Reaction volume was usually 10 µL. Unfolding of the protein was monitored by measuring the fluorescence of the 1,8-anilinonaphthalene sulfonate (ANS), at 50–100 µM. The samples were heated at a rate of 1 °C/min. The samples were excited with 365 nm UV light and ANS fluorescence emission was registered at 460 nm light. Data analysis was performed as previously described [29].

### 3.5. General

All chemicals and solvents were purchased from Aldrich (Dorset, UK) or Alfa-Aesar (Heysham, UK) and used without further purification. All reactions were carried out under a positive pressure of nitrogen or argon in flame or oven-dried glassware. Ethanol was dried over Mg/I_2_; pyridine was dried over KOH pellets; all the other solvents were dried on a MBRAWN SPS-800 apparatus.

Thin layer chromatography (TLC) analysis was performed on silica pre-coated SIL G-25 UV_254_ sheets (layer: 0.25 mm silica gel with fluorescent indicator UV_254_, Alugram, Aldrich, Dorset, UK). Compounds were visualized by UV light (UV lamp, model UVGL-58, Mineralight LAMP, Multiband UV-254/365 nm) and stained with potassium permanganate. Flash column chromatography was carried out on silica gel (40–63 μm, Fluorochem, Hadfield, UK) or, where indicated, on basic alumina (Brockmann I, Sigma-Aldrich). Melting points were measured with an Electrothermal 9100 capillary melting point apparatus and are uncorrected.

Fourier Transform infra-red spectra (FT-IR) were acquired on a Perkin Elmer Paragon 1000 FT spectrometer. Absorption maxima are reported in wavenumbers (cm^−1^).

Unless otherwise stated, ^1^H-NMR spectra were measured at room temperature (298 K) on a Bruker DPX 400 (^1^H = 400 MHz) and Bruker Avance 300 (^1^H = 300.1 MHz) instruments. Deuterated solvents were used and ^1^H-NMR chemical shifts were internally referenced to CHCl_3_ (7.26 ppm) in chloroform-d_1_ solution. Chemical shifts are expressed as δ in unit of ppm. 

^13^C-NMR spectra were recorded in the same conditions and in the same solvents using the PENDANT sequence mode on a Bruker DPX 400 (^13^C = 100 MHz). Data processing was carried out using TOPSPIN 2 NMR version (Bruker UK, Ltd, Coventry, UK). In ^1^H-NMR assignment the multiplicity used is indicated by the following abbreviations: s = singlet, d = doublet, dd = doublet of doublets, t = triplet, q = quartet, m = multiplet, brs = broad singlet. Signals of protons and carbons were assigned, as far as possible, by using the following two-dimensional NMR spectroscopy techniques: [^1^H-^1^H] COSY, [^1^H-^13^C] COSY (HSQC: Heteronuclear Single Quantum Coherence) and long range [^1^H-^13^C] COSY (HMBC: Heteronuclear Multiple Bond Connectivity). 

Mass spectrometry (electrospray mode, ES; chemical ionization mode, CI) were recorded on a high performance orthogonal acceleration reflecting TOF mass spectrometer operating in positive and negative mode, coupled to a Waters 2975 HPLC.

### 3.6. Synthesis

#### 3.6.1. General Procedure for the alkylation of **3**

To a stirred solution of **3** (1 equiv.) in dry DMF (1 vol.) under N_2_ was added K_2_CO_3_ (2 equiv.) and the alkyl halide (1.1 equiv.). The resulting solution was stirred for 16 h at room temperature before being partitioned between ethyl acetate (1 vol.) and water (0.5 vol.). The organic layer was washed with water (0.5 vol.), brine (0.5 vol.), dried (MgSO_4_), filtered and the solvent removed *in vacuo* to give the desired product which was used without further purification.

*Methyl 3,5-dichloro-4-ethoxybenzoate* (**4f**). Prepared from compound **2** (1.3 g, 5.9 mmol), K_2_CO_3_ (1.6 g, 11.7 mmol) and iodoethane (522 μL, 6.5 mmol) in DMF (10 mL). The product was obtained as a brown oil (1.4 g, 5.6 mmol, 96%). *ν*_max_ cm^−1^ (NaCl, thin layer) 2994, 1701, 1652, 1556, 1147, 854; δ_H_ (CDCl_3_, 400 MHz) 7.91 (2H, s, ArH), 4.09 (2H, q, *J* = 7.0 Hz, CH_2_), 3.85 (3H, s, CH_3_) and 1.41 (3H, t, *J* = 7.0 Hz, CH_3_); δ_C_ (CDCl_3_, 100 MHz) 164.7 (C), 155.5 (C), 130.2 (CH), 129.8 (C), 126.9 (C), 70.0 (CH_2_), 52.6 (CH_3_) and 15.5 (CH_3_); *m/z* (ES)^+^: 249.35 [(M + H)^+^, 100%]. 

*Methyl 4-propoxy-3,5-dichlorobenzoate* (**4g**). Prepared from **2** (500 mg, 2.3 mmol), K_2_CO_3_ (630 mg, 4.6 mmol) and iodopropane (243 μL, 2.5 mmol) in DMF (10 mL). The product was obtained as a yellow-brown oil (472 mg, 1.8 mmol, 78%). *ν*_max_ cm^−1^ (NaCl, thin layer) 2954 2880, 1733, 1690, 1592, 1556, 1462, 1288, 1138, 986; δ_H_ (CDCl_3_, 400 MHz) 7.90 (2H, s, ArH), 3.97 (2H, t, *J* = 6.6 Hz, CH_2_), 3.84 (3H, s, CH_3_), 1.82 (2H, app. sextet, *J* = 7.0 Hz, CH_2_) and 1.02 (3H, t, *J* = 7.5 Hz, CH_3_); δ_C_ (CDCl_3_, 100 MHz) 164.2 (C), 155.0 (C), 130.6 (CH), 130.1 (C), 127.3 (C), 76.0 (CH_2_), 53.6 (CH_3_), 23.8 (CH_2_) and 10.8 (CH_3_); *m/z* (ES)^+^: 263.24 [(M + H)^+^, 100%].

*Methyl 4-butoxy-3,5-dichlorobenzoate* (**4h**). Prepared from **2** (500 mg, 2.3 mmol), K_2_CO_3_ (630 mg, 4.6 mmol) and iodobutane (283 μL, 2.5 mmol) in DMF (10 mL). The product was obtained as a brown oil (578 mg, 2.1 mmol, 91%). *ν*_max_ cm^−1^ (NaCl, thin layer) 2959 (ArC-H), 1729, 1642, 1556, 1435, 1284, 1137, 987; δ_H_ (CDCl_3_, 400 MHz) 7.90 (2H, s, ArH), 4.01 (2H, t, *J* = 6.8 Hz, CH_2_), 3.84 (3H, s, CH_3_), 1.81–1.76 (2H, m, CH_2_), 1.50 (2H, app. sextet, *J* = 7.5 Hz, CH_2_) and 0.93 (3H, t, *J* = 7.5 Hz, CH_3_); δ_C_ (CDCl_3_, 100 MHz) 164.7 (C), 155.6 (C), 130.3 (CH), 129.7 (C), 126.9 (C), 73.8 (CH_2_), 52.6 (CH_3_), 32.11 (CH_2_), 19.04 (CH_2_) and 13.8 (CH_3_); *m/z* (ES)^+^: 277.06 [(M + H)^+^, 100%]. 

#### 3.6.2. General Procedure for Ester Hydrolysis

The ester (1 equiv.) and sodium hydroxide (1.2 equiv.) were heated at reflux in a solution of methanol (1 vol.) and water (1 vol.) until the methyl ester was consumed by TLC (4–6 h). The methanol was removed *in vacuo* and the aqueous fraction acidified with 2 M HCl. The resulting precipitate was extracted with ethyl acetate (3 × 1 vol.) and the organic layers combined and washed with brine (0.5 vol.), dried (MgSO_4_), filtered and the solvent removed to yield the desired acid.

*3,5-Dichloro-4-ethoxybenzoic acid* (**5f**). Prepared from methyl 4-ethoxy-3,5-dichlorobenzoate (500 mg, 2.0 mmol) in MeOH/water (10 mL) and NaOH (96 mg, 2.4 mmol). The desired product was obtained as an off-white solid (1.8 g, 7.7 mmol, 75%). Mp 179–180 °C; *ν*_max_ cm^−1^ (KBr) 3225, 2104, 1635, 1206; δ_H_ (CDCl_3_, 400 MHz) 8.12 (2H, s, ArH), 4.28 (2H, q, *J* = 6.9 Hz, CH_2_), 1.57 (3H, t, *J* = 6.9 Hz, CH_3_); δ_C_ (CDCl_3_, 100 MHz) 169.6 (C), 156.3 (C), 130.8 (CH), 130.0 (C), 125.9 (C), 70.2 (CH_2_), 15.5 (CH_3_); *m/z* (ES)^−^ 232.97 [(M−H)^−^, 100%]; HRMS (ES^−^) [Found: (M-H)^−^, 232.9767, C_9_H_7_O_3_Cl_2_ requires 232.9772].

*4-Propoxy-3,5-dichlorobenzoic acid* (**5g**). Prepared from methyl 4-propoxy-3,5-dichlorobenzoate (400 mg, 1.5 mmol) in MeOH/water (10 mL) and NaOH (72 mg, 1.8 mmol). The product was obtained as a white solid (347 mg, 1.4 mmol, 93%). Mp 125–126 °C; *ν*_max_ cm^−1^ (KBr) 2937, 1699, 1558, 1493, 1385, 1077, 906, 768; δ_H_ (CDCl_3_, 400 MHz) 7.97 (2H, s, ArH), 4.00 (2H, t, *J* = 6.6 Hz, CH_2_), 1.83 (2H, app. sextet, *J* = 7.1 Hz, CH_2_), 1.03 (3H, t, *J* = 7.6 Hz, CH_3_); δ_C_ (CDCl_3_, 100 MHz) 169.4, 156.4, 129.9, 125.9, 75.7, 23.4, 10.4; *m/z* (ES)^−^ 247.23 [(M−H)^−^, 100%]; HRMS (ES^−^) [Found: (M−H)^−^, 246.9924, C_10_H_9_O_3_Cl_2_ requires 246.9929] 

*4-Butoxy-3,5-dichlorobenzoic acid* (**5h**). Prepared from methyl 4-butoxy-3,5-dichlorobenzoate (500 mg, 1.8 mmol) in MeOH/water (10 mL) and NaOH (86 mg, 2.2 mmol). The product was obtained as a yellow solid (472 mg, 1.8 mmol, 99%). Mp 98–99 °C; *ν*_max_ cm^−1^ (KBr) 2955, 1682, 1214, 1557, 1388, 1056, 810; δ_H_ (CDCl_3_, 400 MHz) 7.91 (2H, s, ArH), 4.04 (2H, t, *J* = 6.6 Hz, CH_2_), 1.8–1.7 (2H, m, CH_2_), 1.49 (2H, app. sextet, *J* = 7.0 Hz, CH_2_), 0.94 (3H, t, *J* = 7.4 Hz, CH_3_); δ_C_ (CDCl_3_, 100 MHz) 169.3 (C), 156.4 (C), 130.8 (CH), 130.2 (C), 125.9 (C), 73.8 (CH_2_), 32.1 (CH_2_), 19.03 (CH_2_), 13.8 (CH_3_); *m/z* (ES)^−^ 261.02 [(M–H)^−^, 100%]; HRMS (ES^−^) [Found: (M–H)^−^, 261.0078, C_11_H_11_O_3_Cl_2_ requires 261.0085].

#### 3.6.3. General Procedure for Synthesis of Acid Chlorides **1a**–**1**

To a stirred solution of the benzoic acid (synthesised or commercially available) (1 equiv.) in DCM (1 vol.), under N_2_, was added a solution of oxalyl chloride (2 equiv.) in DCM (1 vol.). A drop of dry DMF was added and the resulting solution stirred at room temperature for 90 min. The solvent was removed *in vacuo* and the resulting acid chloride used immediately without purification or characterisation.

#### 3.6.4. General Procedure for Sodium Thiocyanate Coupling Reaction

To a solution of the acid chloride (1 equiv.) in dry acetone (1 vol.) under N_2_, was added sodium thiocyanate (1 equiv.). The resulting suspension stirred at room temperature for 30 min before being cooled to 0 °C. A solution of the amine (1 equiv.) in dry acetone (1 vol.) was added and the resulting suspension allowed to warm to room temperature and stirred for 16 h. The reaction was filtered through Celite and the filtrate concentrated to give the crude product. The product was purified by column chromatography (1–20% methanol-DCM). The purified product was dissolved in acetone and 2 M HCl in diethyl ether (1 equiv.) added slowly. The resulting precipitate was filtered and recrystallised from ethanol to afford the pure HCl salts.

*3,5-Dibromo-N-((4-(5-(dimethylamino)pentanamido)phenyl)carbamothioyl)-4-methoxybenzamide hydrochloride* (**Tenovin-36**). Prepared from 3,5-dibromo-4-methoxybenzoyl chloride (85 mg, 0.26 mmol) and sodium thiocyanate (21 mg, 0.26 mmol) in dry acetone (3 mL) followed by addition of *N*-(4-aminophenyl)-5-(dimethylamino)pentanamide (61 mg, 0.26 mmol) in acetone (3 mL). The crude material was purified by column chromatography (1–20% MeOH-DCM) and this material subsequently converted to the HCl salt to afford the product as a yellow solid (40 mg, 0.06 mmol, 23%). Mp 159–160 °C; *ν*_max_ cm^−1^ (KBr) 2924, 2857, 1669, 1606, 1541, 1495, 1405, 1262, 1094, 824; δ_H_ (DMSO-*d_6_*, 400 MHz) 12.41 (1H, s, NH), 11.85 (1H, s, NH), 10.21 (1H, s, NH), 9.75 (1H, br. s, NH^+^), 8.34 (2H, s, ArH), 7.69 (4H, AA’BB’, *J* = 8.8, 29.0 Hz, ArH), 3.96 (3H, s, CH_3_), 3.14 (2H, m, CH_2_), 2.83 (6H, d, *J* = 4.8 Hz, (CH_3_)_2_), 2.48 (2H, m, CH_2_), 1.74 (4H, m, (CH_2_)_2_); δ_C_ (DMSO-*d_6_*, 400 MHz) 178.5 (C), 170.8 (C), 164.9 (C), 156.9 (C), 137.5 (C), 133.2 (CH), 132.7 (C), 130.7 (C), 124.7 (CH), 119.0 (CH), 117.4 (C), 60.6 (CH_3_), 56.1 (CH_2_), 41.9 (CH_3_), 35.5 (CH_2_), 23.2 (CH_2_) and 22.0 (CH_2_); *m/z* (ES)^+^ 586.98 [((M−HCl)+H)^+^, 100%].

*N-((4-(5-(Dimethylamino)pentanamido)phenyl)carbamothioyl)-3,4,5-trimethoxybenzamide hydro-chloride* (**Tenovin-37**). Prepared from 3,4,5-trimethoxybenzoyl chloride (53 mg, 0.23 mmol) and sodium thiocyanate (19 mg, 0.23 mmol) in dry acetone (3 mL) followed by addition of *N*-(4-aminophenyl)-5-(dimethylamino)pentanamide (54 mg, 0.23 mmol) in acetone (3 mL). The crude material was purified by column chromatography (1–20% MeOH-DCM) and this material subsequently converted to the HCl salt to afford the product as a yellow solid (36.3 mg, 0.06 mmol, 30%). Mp 115–117 °C; *ν*_max_ cm^−1^ (KBr) 2925, 2858, 1670, 1642, 1495, 1405, 1340, 1261, 1125, 1050, 824; δ_H_ (DMSO-*d_6_*, 300 MHz) 12.65 (1H, s, NH), 11.56 (1H, s, NH), 10.15 (1H, s, NH), 9.82 (1H, br. s, NH^+^), 7.62 (4H, AA’BB’, *J* = 9.0, 24.4 Hz, ArH), 7.38 (2H, s, ArH), 3.88 (6H, s, (CH_3_)_2_), 3.75 (3H, s, CH_3_), 3.06 (2H, m, CH_2_), 2.75 (6H, d, *J* = 4.9 Hz, (CH_3_)_2_), 2.39 (2H, t, *J* = 6.5 Hz, CH_2_), 1.65 (4H, m, (CH_2_)_2_); δ_C_ (DMSO-*d_6_*, 100 MHz) 178.9 (C), 170.7 (C), 167.4 (C), 152.5 (C), 141.5 (C), 137.4 (C), 132.8 (C), 126.7 (C), 124.7 (CH), 119.0 (CH), 106.5 (CH), 60.1 (CH_3_), 56.6 (CH_2_), 56.1 (CH_3_), 41.9 (CH_3_), 35.5 (CH_2_), 23.2 (CH_2_), 23.2 (CH_2_), 21.9 (CH_2_); *m/z* (ES)^+^ 489.18, [((M−HCl)+H)^+^, 100%]; HRMS (ES)^+^ [Found: ((M−HCl)+H)^+^, 489.2172, C_24_H_33_ClN_4_O_5_S requires 489.2166].

*N-(4-(5-(dimethylamino)pentanamido)phenylcarbamothioyl)-4-methoxy-3,5-dimethylbenzamide hydrochloride* (**Tenovin-38**). Prepared from 4-methoxy-3,5-dimethylbenzoyl chloride (110 mg, 0.6 mmol) and sodium thiocyanate (45 mg, 0.6 mmol) in dry acetone (3 mL) followed by addition of *N*-(4-aminophenyl)-5-(dimethylamino)pentanamide (130 mg, 0.6 mmol) in acetone (3 mL). The crude material was purified by column chromatography (1–20% MeOH-DCM) and this material subsequently converted to the HCl salt. The product was obtained as a brown solid (74 mg, 0.15 mmol, 25%). Mp 190–192 °C; *ν*_max_ cm^−1^ (KBr) 2925, 2854, 1667, 1604, 1515, 1337, 1307, 1166, 1007, 740; δ_H_ (*d*_6_-DMSO, 400 MHz) 12.64 (1H, s, NH), 11.36 (1H, s, NH), 10.20 (1H, s, NH), 9.91 (1H, br. s, NH^+^), 7.79 (2H, s, ArH), 7.69 (2H, d, *J* = 8.4 Hz, ArH), 7.63 (2H, d, *J* = 8.4 Hz, ArH), 3.77 (3H, s, CH_3_), 3.15–3.08 (2H, m, CH_2_), 2.79 (6H, s, (CH_3_)_2_), 2.47–2.41 (2H, m, CH_2_), 2.33 (6H, s, (CH_3_)_2_), 1.76–1.69 (4H, m, (CH_2_)_2_); δ_C_ (*d*_6_-DMSO, 100 MHz) 178.9 (C), 170.7 (C), 167.7 (C), 160.7 (C), 137.3 (C), 132.8 (C), 130.6 (C), 129.5 (CH), 126.9 (C), 124.7 (CH), 118.9 (CH), 59.4 (CH_3_), 56.2 (CH_2_), 42.0 (CH_3_), 35.5 (CH_2_), 23.3 (CH_2_), 21.9 (CH_2_), 15.8 (CH_3_); *m/z* (ES)^+^ 457.03 [((M−HCl)+H)^+^, 100%]; HRMS (ES)^+^ [Found: ((M−HCl)+H)^+^, 457.2265, C_24_H_33_N_4_O_3_S requires 457.2273].

*N-((4-(5-(dimethylamino)pentanamido)phenyl)carbamothioyl)-3,5-difluoro-4-methoxybenzamide hydrochloride* (**Tenovin-39**). Prepared from 3,5-difluoro-4-methoxybenzoyl chloride (132 mg, 0.64 mmol) and sodium thiocyanate (52 mg, 0.64 mmol) in dry acetone (3 mL) followed by addition of *N*-(4-aminophenyl)-5-(dimethylamino)pentanamide (151 mg, 0.64 mmol) in acetone (3 mL). The crude material was purified by column chromatography (1–20% MeOH-DCM) and this material subsequently converted to the HCl salt to afford the product as an off-white solid (134 mg, 0.27 mmol, 42%). Mp 153–154 °C; *ν*_max_ cm^−1^ (KBr) 2924, 2859, 1659, 1608, 1544, 1495, 1451, 1404, 1262, 1180, 1049, 824; δ_H_ (*d*_6_-DMSO, 400 MHz) 12.33 (1H, s, NH), 11.54(1H, s, NH), 10.19 (2H, br. s., (NH)_2_), 7.81 (2H, d, *J* = 8.6 Hz, ArH), 7.62 (2H, d, *J* = 6.8 Hz, ArH), 7.53 (2H, d, *J* = 6.8 Hz, ArH), 4.08 (3H, s, CH_3_), 3.00 (2H, app. br. s., CH_2_), 2.68 (6H, s, (CH_3_)_2_), 2.35 (2H, app. br. s., CH_2_) and 1.62 (4H, app. br. s., (CH_2_)_2_); δ_C_ (*d*_4_-CD_3_OD, 100 MHz) 180.5 (C), 173.7 (C), 167.0 (C), 157.2 (C), 155.2 (C), 138.3 (C), 135.2 (C), 127.8 (C), 125.9 (CH), 121.1 (CH), 114.1 (CH), 58.7 (CH_2_), 53.6 (CH_3_), 43.5 (CH_3_), 36.7 (CH_2_), 25.2 (CH_2_), 23.1 (CH_2_); *m/z* (ES)^+^ 465.03 [((M−HCl)+H)^+^, 100%]; HRMS (ES)^+^ [Found: ((M−HCl)+H)^+^, 465.1760, C_22_H_27_N_4_O_3_F_2_S requires 465.1772].

*N-((4-(5-(dimethylamino)pentanamido)phenyl)carbamothioyl)-3,5-difluoro-4-ethoxybenzamide hydrochloride* (**Tenovin-40**). Prepared from 3,5-difluoro-4-ethoxybenzoyl chloride (150 mg, 0.68 mmol) and sodium thiocyanate (55 mg, 0.68 mmol) in dry acetone (3 mL) followed by addition of *N*-(4-aminophenyl)-5-(dimethylamino)pentanamide (160 mg, 0.68 mmol) in acetone (3 mL). The crude material was purified by column chromatography (1–20% MeOH-DCM) and this material subsequently converted to the HCl salt to afford the product as an off-white solid (124 mg, 0.24 mmol, 35%). Mp 206–207 °C; *ν*_max_ cm^−1^ (KBr) 2926, 2860, 1674, 1606, 1512, 1495, 1436, 1405, 1263, 1135, 1049, 874; δ_H_ (*d*_6_-DMSO, 400 MHz) 12.34 (1H, s, NH), 11.56 (1H, br. s, NH), 10.11 (2H, s, NH), 9.70 (2H, br. s., NH^+^), 7.83 (2H, d, *J* = 9.2 Hz, ArH), 7.64 (2H, d, *J* = 7.9 Hz, ArH), 7.56 (2H, d, *J* = 7.9 Hz, ArH), 4.31 (2H, app quartet, *J* = 4.3 Hz, CH_2_), 3.08-2.99 (2H, m, CH_2_), 2.72 (6H, app. s, (CH_3_)_2_), 2.38 (2H, app. t., *J* = 6.3 Hz, CH_2_), 1.71–1.56 (4H, m, (CH_2_)_2_) and 1.32 (3H, t, *J* = 7.0 Hz, CH_3_); δ_C_ (*d*_4_-CD_3_OD, 100 MHz) 180.4 (C), 173.5 (C), 167.0 (C), 157.6 (C), 155.7 (C), 138.3 (C), 135.2 (C), 128.0 (C), 125.9 (CH), 121.1 (CH), 113.9 (CH), 58.7 (CH_2_), 43.5 (CH_3_), 36.7 (CH_2_), 30.8 (CH_2_), 25.2 (CH_2_), 23.1 (CH_2_), 15.8 (CH_3_); *m/z* (ES)^+^ 478.90 [((M−HCl)+H)^+^, 100%]; HRMS (ES)^+^ [Found: ((M−HCl)+H)^+^, 479.1940, C_23_H_29_N_4_O_3_F_2_S requires 479.1928].

*3,5-Dichloro-N-(4-(5-(dimethylamino)pentanamido)phenylcarbamothioyl)-4-ethoxybenzamide hydrochloride* (**Tenovin-41**). Prepared by the reaction of 3,5-dichloro-4-ethoxybenzoyl chloride (216 mg, 0.86 mmol) and sodium thiocyanate (69 mg, 0.86 mmol) in dry acetone (6 mL) followed by addition of a solution of *N*-(4-aminophenyl)-5-(dimethylamino)pentanamide (202 mg, 0.86 mmol) in dry acetone (6 mL) using general method F. The product was further purified by conversion to the HCl salt by the addition of 2M HCl in diethyl ether. The desired product was obtained as a brown sticky oil (42 mg, 0.08 mmol, 9.5%). *ν*_max_ cm^−1^ (NaCl, thin layer) 2965, 2359.3, 1651, 1048, 1025, 998, 765; δ_H_ (CDCl_3_, 400 MHz) 12.29 (1H, s, NH), 11.68 (1H, s, NH), 10.21 (1H, s, NH), 8.08 (2H, s, ArH), 7.79–7.46 (4H, m, ArH), 4.19–4.06 (2H, m, CH_2_), 3.06–2.93 (2H, m, CH_2_), 2.69 (6H, s, (CH_3_)_2_), 2.36–2.24 (2H, m, CH_2_), 1.71–1.65 (2H, m, CH_2_), 1.41–1.28 (2H, m, CH_2_), 1.23–1.17 (3H, m, CH_3_); δ_C_ (*d*_4_-CD_3_OD, 100 MHz) 180.4 (C), 173.5 (C), 167.0 (C), 156.7 (C), 138.2 (C), 135.2 (C), 131.1 (C), 130.9 (C), 130.4 (CH), 125.9 (CH), 121.2 (CH), 71.2 (CH_2_), 58.8 (CH_2_), 43.5 (CH_3_), 36.6 (CH_2_), 25.3 (CH_2_), 23.1 (CH_2_), 15.8 (CH_3_); *m/z* (ES)^+^ 510.94 [((M−HCl)+H)^+^, 100%]; HRMS (ES)^+^ [Found: ((M−HCl)+H)^+^, 511.1337, C_23_H_29_N_4_O_3_SCl_2_ requires 511.1337].

*3,5-Dichloro-N-((4-(5-(dimethylamino)pentanamido)phenyl)carbamothioyl)-4-propoxybenzamide hydrochloride* (**Tenovin-42**). Prepared from 3,5-dichloro-4-propoxybenzoyl chloride (160 mg, 0.60 mmol) and sodium thiocyanate (49 mg, 0.60 mmol) in dry acetone (3 mL) followed by addition of *N*-(4-aminophenyl)-5-(dimethylamino)pentanamide (142 mg, 0.60 mmol) in acetone (3 mL). The crude material was purified by column chromatography (1–20% MeOH-DCM) and this material subsequently converted to the HCl salt to afford the product as an off-white solid (73 mg, 0.13 mmol, 21%). Mp 167–168 °C; *ν*_max_ cm^−1^ (KBr) 2927, 2861, 1674, 1636, 1606, 1539, 1473, 1452, 1265, 1148, 1051, 873; δ_H_ (*d*_6_-DMSO, 400 MHz) 12.31 (1H, s, NH), 11.70 (1H, s, NH), 10.23 (1H, s, NH), 10.13 (1H, s, NH^+^), 8.09 (2H, s, ArH), 7.65 (2H, d, *J* = 8.9 Hz, ArH), 7.56 (2H, d, *J* = 8.9 Hz, ArH), 4.04 (2H, t, *J* = 6.4 Hz, CH_2_), 3.02–2.96 (2H, m, CH_2_), 2.72 (6H, d, *J* = 5.2 Hz, (CH_3_)_2_), 2.38–2.32 (2H, m, CH_2_), 1.86–1.75 (2H, m, CH_2_), 1.70–1.60 (4H, m, (CH_2_)_2_), 1.03 (3H, t, *J* = 7.6 Hz, CH_3_); δ_C_ (*d*_6_-DMSO, 100 MHz) 170.5 (C), 165.5 (C), 154.4 (C), 150.5 (C), 135.4 (C), 132.7 (C), 129.5 (C), 129.2 (CH), 128.6 (C), 120.2 (CH), 119.6 (CH), 75.4 (CH_2_), 56.2 (CH_2_), 42.0 (CH_3_), 35.4 (CH_2_), 23.3 (CH_2_), 22.9 (CH_2_), 21.9 (CH_2_), 10.6 (CH_3_); *m/z* (ES)^+^ 525.09 [((M−HCl)+H)^+^, 100%]; HRMS (ES)^+^ [Found: ((M−HCl)+H)^+^, 525.1509, C_24_H_31_N_4_O_3_SCl_2_ requires 525.1494]. 

*4-Butoxy-3,5-dichloro-N-((4-(5-(dimethylamino)pentanamido)phenyl)carbamothioyl)benzamide hydrochloride* (**Tenovin-43**). Prepared from 3,5-dichloro-4-butoxybenzoyl chloride (28 mg, 0.10 mmol) and sodium thiocyanate (8 mg, 0.10 mmol) in dry acetone (2 mL) followed by addition of *N*-(4-aminophenyl)-5-(dimethylamino)pentanamide (24 mg, 0.10 mmol) in acetone (2 mL). The crude material was purified by column chromatography (1–20% MeOH-DCM) and this material subsequently converted to the HCl salt to afford the product as a cream solid (31 mg, 0.05 mmol, 51%). Mp 174–176 °C; *ν*_max_ cm^−1^ (KBr) 2959, 2927, 2858, 1674, 1606, 1545, 1495, 1405, 1264, 1151, 1050, 876; δ_H_ (*d*_6_-DMSO, 400 MHz) 12.25 (1H, s, NH), 11.64 (1H, s, NH), 10.08 (1H, s, NH), 9.66 (1H, s, NH^+^), 8.03 (2H, s, ArH), 7.59 (2H, d, *J* = 8.8 Hz, ArH), 7.50 (2H, d, *J* = 8.8 Hz, ArH), 4.01 (2H, t, *J* = 6.3 Hz, CH_2_), 3.04–2.93 (2H, m, CH_2_), 2.69 (6H, d, *J* = 4.5 Hz, (CH_3_)_2_), 2.32 (2H, app. t, *J* = 6.7 Hz, CH_2_), 1.71 (2H, app. sextet, *J* = 8.0 Hz, CH_2_), 1.66–1.50 (4H, m, (CH_2_)_2_), 1.45 (2H, app. sextet, *J* = 7.3 Hz, CH_2_), 0.89 (3H, t, *J* = 7.4 Hz, CH_3_); δ_C_ (*d*_6_-DMSO, 100 MHz) 179.5 (C) 170.5 (C), 165.6 (C), 154.4 (C), 135.3 (C), 132.5 (C), 129.5 (C), 129.2 (CH), 128.6 (C), 120.3 (CH), 119.6 (CH), 73.6 (CH_2_), 56.2 (CH_2_), 42.1 (CH_3_), 35.4 (CH_2_), 31.5 (CH_2_), 23.3 (CH_2_), 21.8 (CH_2_), 18.5 (CH_2_), 13.6 (CH_3_); *m/z* (ES)^+^ 539.09 [((M−HCl)+H)^+^, 100%]; HRMS (ES)^+^ [Found: ((M−HCl)+H)^+^, 539.1655, C_25_H_33_N_4_O_3_SCl_2_ requires 539.1655].

*4-Butoxy-N-((4-(5-(dimethylamino)pentanamido)phenyl)carbamothioyl)benzamide hydrochloride* (**Tenovin-44**). Prepared from 4-butoxybenzoyl chloride (45 mg, 0.21 mmol) and sodium thiocyanate (17 mg, 0.21 mmol) in dry acetone (3 mL) followed by addition of *N*-(4-aminophenyl)-5-(dimethylamino)pentanamide (49 mg, 0.21 mmol) in acetone (3 mL). The crude material was purified by column chromatography (1–20% MeOH-DCM) and this material subsequently converted to the HCl salt to afford the product as a brown solid (72 mg, 0.14 mmol, 68%). Mp 160–161 °C; *ν*_max_ cm^−1^(KBr) 2956, 2927, 2861, 1670, 1606, 1546, 1495, 1408, 1251, 1190, 1050, 875; δ_H_ (*d*_6_-DMSO, 400 MHz) 12.63 (1H, s, NH), 11.33 (1H, s, NH), 10.43 (1H, s, NH^+^), 10.27 (1H, s, NH), 7.98 (2H, d, *J* = 8.8 Hz, ArH), 7.66 (2H, d, *J* = 8.8 Hz, ArH), 7.57 (2H, d, *J* = 8.8 Hz, ArH), 7.04 (2H, d, *J* = 8.8 Hz, ArH), 4.06 (2H, t, *J* = 6.5 Hz, CH_2_), 3.09-3.00 (2H, m, CH_2_), 2.70 (6H, d, *J* = 5.0 Hz, (CH_3_)_2_), 2.38 (2H, app. t, *J* = 6.7 Hz, CH_2_), 1.75–1.57 (6H, m, (CH_2_)_3_), 1.43 (2H, app. sextet, *J* = 7.6 Hz, CH_2_), 0.93 (3H, t, *J* = 7.5 Hz, CH_3_); δ_C_ (*d*_4_-CD_3_OD, 100 MHz) 180.8 (C), 173.6 (C), 169.1 (C), 165.0 (C), 138.1 (C), 135.3 (C), 131.5 (CH), 125.8 (CH), 125.2 (C), 121.4 (CH), 115.6 (CH), 69.2 (CH_2_), 58.7 (CH_2_), 43.5 (CH_3_), 36.7 (CH_2_), 32.3 (CH_2_), 25.2 (CH_2_), 23.2 (CH_2_), 20.2 (CH_2_), 14.2 (CH_3_); *m/z* (ES)^+^ 471.04 [((M−HCl)+H)^+^, 100%]; HRMS (ES)^+^ [Found: ((M−HCl)+H)^+^, 471.2429, C_25_H_35_N_4_O_3_S requires 471.2430]. 

^1^H and ^13^C-NMR spectra of all tenovin analogues are included in the electronic Supplementary Information.

## 4. Conclusions

There remains a need for novel approaches to assess the function of the sirtuin family of deacetylases. This is driven by the continuing interest in this important class of proteins. Here we show that ^1^H-NMR methods can be used to follow the deacetylation of a histone H4-based *N*-acetylated substrate. By monitoring the signal corresponding to the methyl group of the *N*-acetyl functional group, reaction of the substrate can be followed. Our previously reported sirtuin inhibitor, tenovin-6, was shown to inhibit this reaction. Detailed studies showed that inhibition by tenovin-6 was dose dependent and that using this system tenovin-6 had an IC_50_ value of 139.2 ± 9.5 µM. Further studies led to the identification of a tenovin-6 analogue that has a high nanomolar IC_50_ value against SIRT2 (tenovin-43). Compounds with this level of potency are rare in the sirtuin inhibitor literature to date [21]. Importantly, the relative activity of tenovins -6 and -43, as judged by our newly developed ^1^H NMR method and the commercially available assay, were in agreement. Tenovin-43 was also shown to inhibit SIRT2 at concentrations where no inhibition of SIRT2 by the current state of the art inhibitor AGK2 was observed. Whilst use of the NMR method is not suitable for the assessment of numerous analogues, the relative activity of inhibitors can be tested. In addition, researchers can use this new method as another means of reassuring themselves that their sirtuin modulators target the relevant enzyme activity. A thermal shift assay was also used with SIRT2 to measure the binding constants of tenovins-6, -43 and AGK2. Whilst the calculated binding constants of the tenovin analogues were in agreement with the order of potencies calculated by the commercially available assay kit and the NMR method, the binding constant for AGK2 was determined to be much larger than expected.

## Supplementary Materials

Supplementary materials can be accessed at: http://www.mdpi.com/1420-3049/17/10/12206/s1.
