# Supplementary material for: Discovery and Validation of SIRT2 Inhibitors Based on Tenovin-6: Use of a 1H-NMR Method to Assess Deacetylase Activity
Source: Molecules. 2012 Oct 18;17(10):12206–24. doi: 10.3390/molecules171012206 (PMC6268290; doi:10.3390/molecules171012206)
Supplement: Supplementary file 1 [file molecules-17-12206-s001.pdf]

Electronic Supplementary Information

Discovery and Validation of SIRT2 Inhibitors Based on Tenovin-6: Use of a ^{1}H-NMR Method to Assess Deacetylase Activity

Figure S1. H1299 cells were treated with tenovin-43 at the indicated concentrations for 16 h together with 40 nM trichostatin A to reduce background due to non-sirtuin HDAC activity. K40-acetylated α-tubulin and total α-tubulin were analysed.

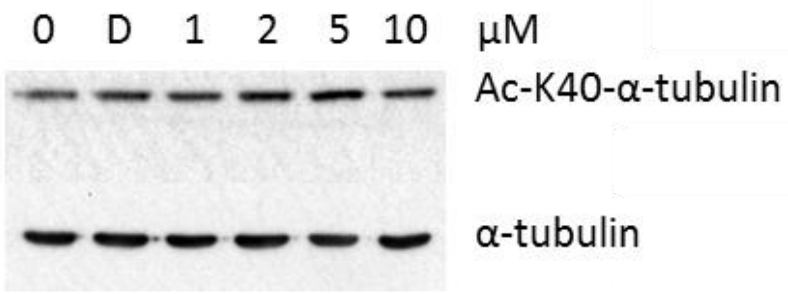

Figure S2. Band-selective ^{1}H, ^{13}C-HMBC of GLGKGGAK(Ac)RHR peptide at room temperature shows a crosspeak for the singlet at 1.86 ppm with the carbonyl carbon at 173.8 ppm which supports the assignment to the acetyl CH_3. Furthermore, the spectrum enabled the identification of the terminal methylene group of the lysine residue (triplet at 3.03 ppm) which also shows a crosspeak with the carbonyl carbon at 173.8 ppm.

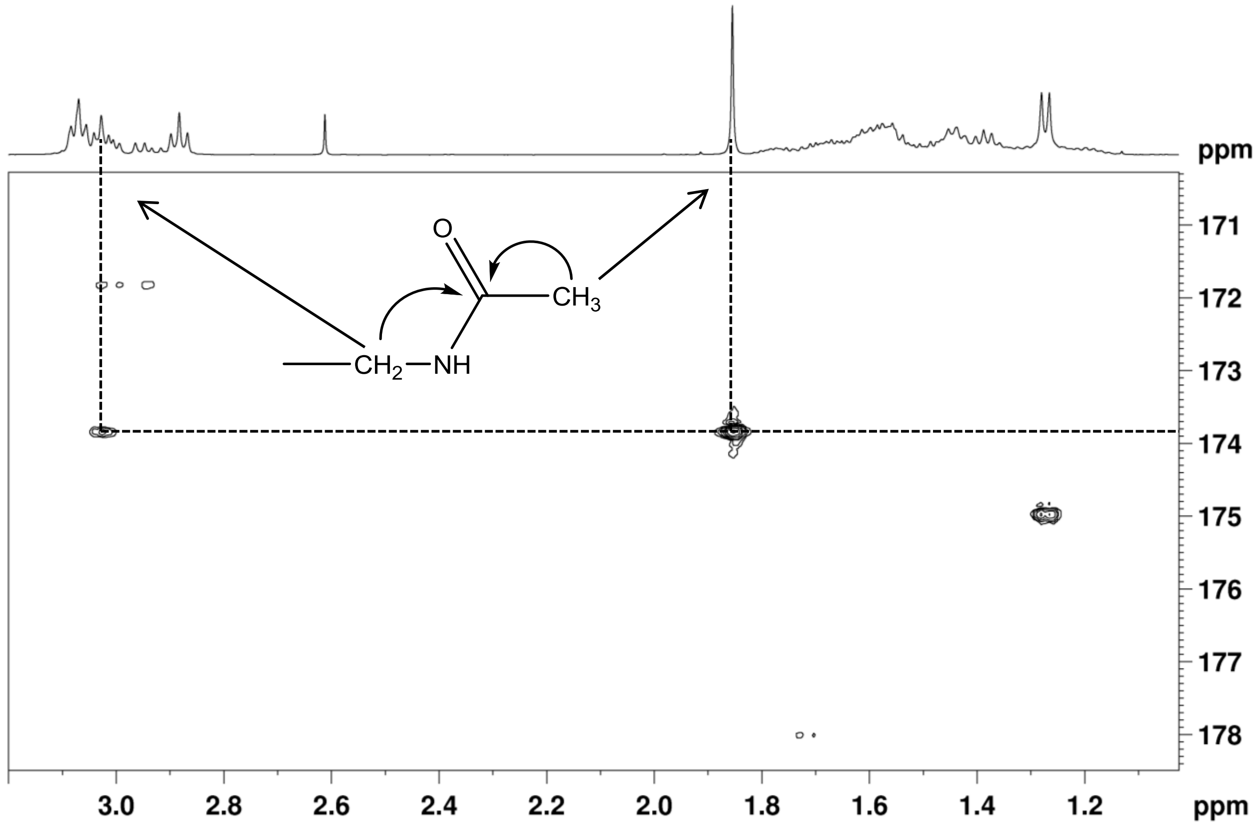

Figure S3. (**a**) 1D gs-TOCSY of GLGKGGAK(Ac)RHR peptide at r.t. The α-proton (4.12 ppm) was selectively excited and used as the starting point for coherence transfer to reveal all resonances of the N-acetylated lysine residue (1.61 (β), 1.20 (γ), 1.39 (δ) and 3.03 (ε) ppm). ^{1}H-NMR spectrum of: (**b**) the assay mixture before adding SIRT2 at r.t.; (**c**) the assay mixture after addition of SIRT2, incubation at 37 °C for 15 min. The dashed lines highlight the lysine resonances which were affected during the reaction.

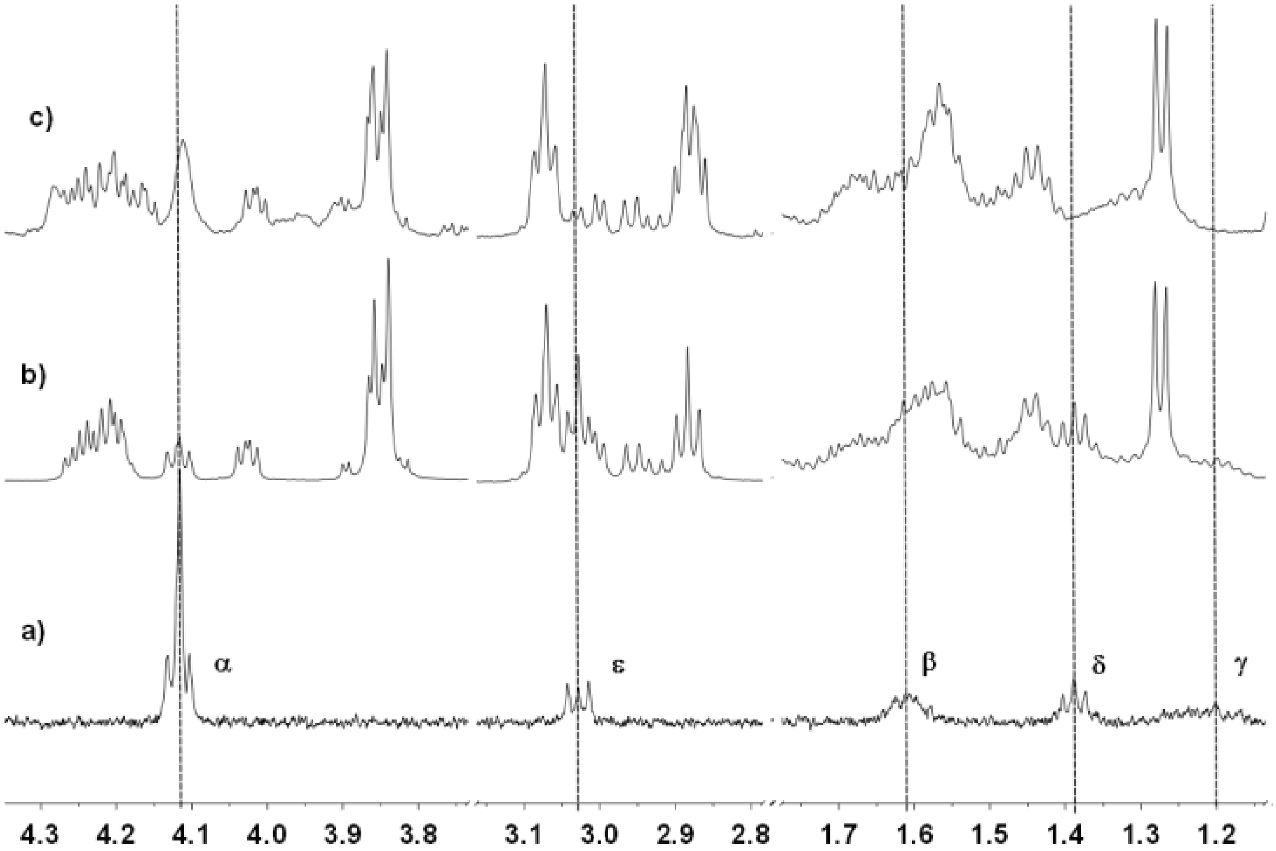

Figure S4. 1D ^{1}H-NMR with double solvent suppression (for both H_2O and Tris buffer) recorded at 37 ℃. Sample contained 1 mM NAD^+, 200 µM peptide, 10 µM SIRT2 in buffer (pH 8). (**a**) Before addition of enzyme. (**b**,**c**) After addition of enzyme and 15 mins. Incubation then addition of authentic *O*-acetyl ADP riboses (as prepared according to Perni *et al.* (2010)).(**d**) ^{1}H-NMR of authentic sample of *O*-acetyl-ADP riboses in buffer (pH 8).

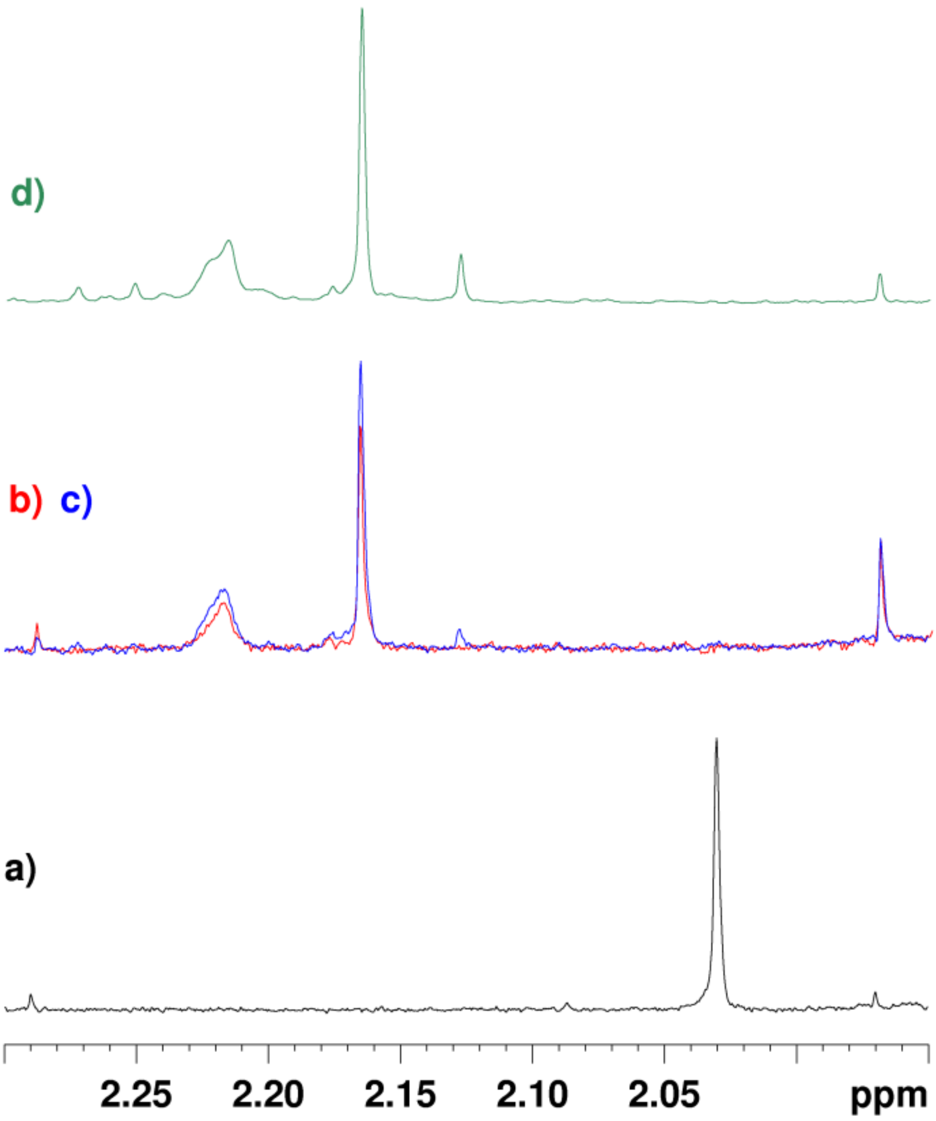

It was also intersting to note the appearance of another acyl peak in this region over time at 1.97 ppm. It has been previously reported[1] that the esters hydrolyse readily in D_2O solution and this was observed in buffer at pH 8.

Figure S5. 1D ^{1}H-NMR with double solvent suppression (for both H_2O and Tris buffer) recorded at 37 ℃. Sample contained 1 mM NAD^+, 200 μM peptide, 10 μM SIRT2 in buffer (pH 8). (**a**) After 20-min Incubation with enzyme. A mixture of 2′-*O*-acetyl ADP ribose and 3′-*O*-acetyl ADP ribose is observed (**b**) After 24 h at room temperature (red) (**c**) After addition of sodium acetate (blue). The peaks directly overlap which implies that this peak is in fact acetate resulting from ester hydrolysis of the reaction products.

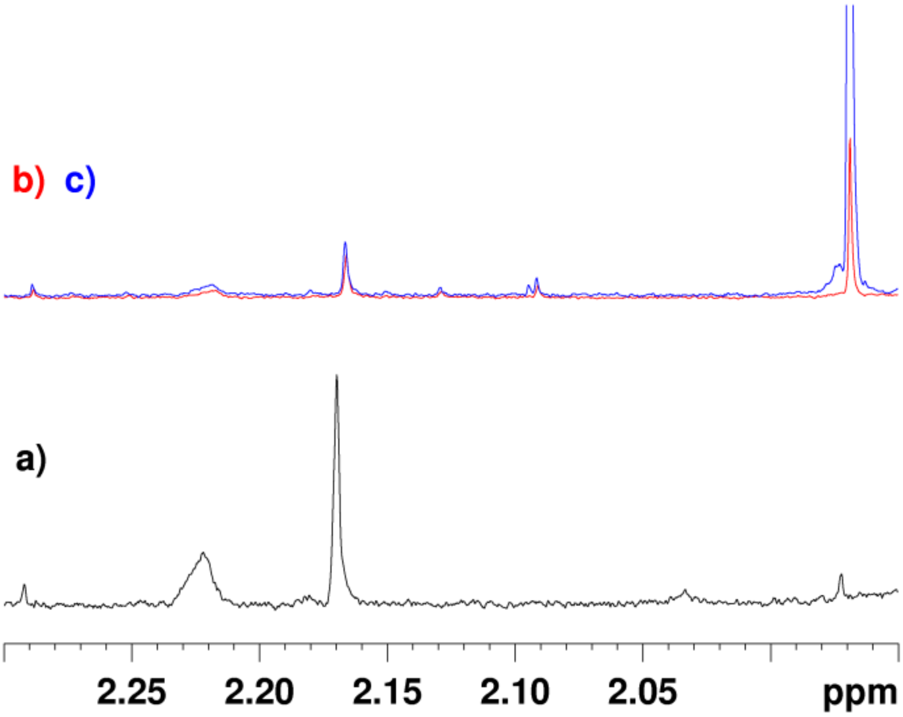

Figure S6. 1D ^{1}H-NMR with double solvent suppression (for both H_2O and Tris buffer) recorded at 37 ℃. Sample contained 1 mM NAD^+, 200 μM peptide, 10 μM SIRT2 in buffer (pH 8). (**a**) After 20-min Incubation with enzyme (shown in blue). The deacetylated peptide is observed. Doping with authentic material (shown in red). The peaks directly overlap which implies that this peaks are in fact from the deacetylated peptide product.

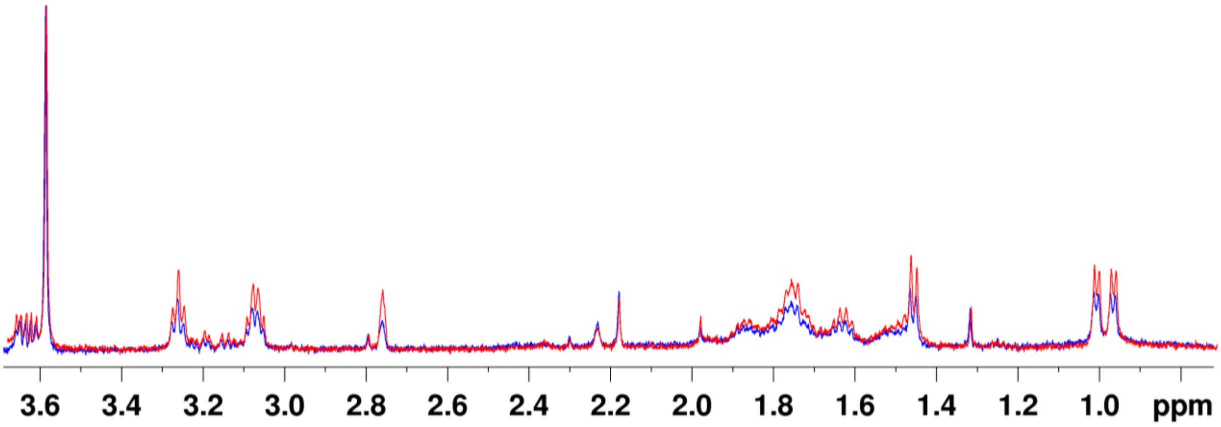

Figure S7. Fluorescence melting curves of SIRT2 at various added concentrations of tenovin-43. The midpoint of the melting temperature (T_m) is shifted towards higher temperature with increasing ligand concentration.

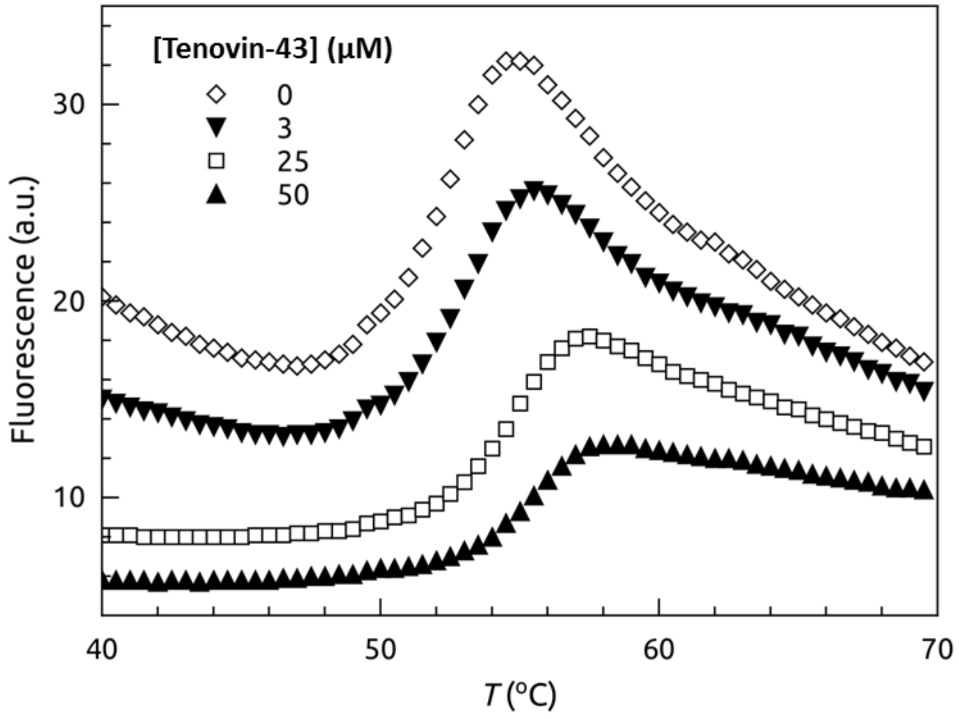

Figure S8. Fluorescence melting curves of SIRT2 at various added concentrations of AGK2. The midpoint of the melting temperature (T_m) remains unchanged.

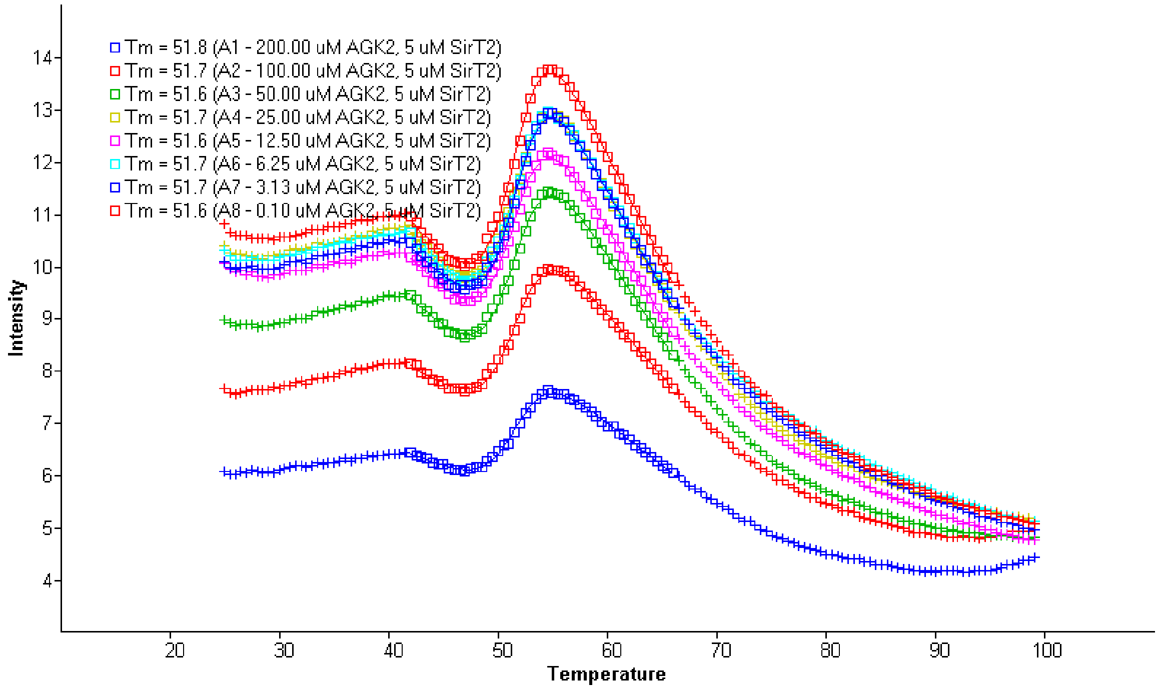

Figure S9. Ligand dosing curves showing the T_m shift dependence of SIRT2 on AGK2 concentration. Datapoints are experimental data obtained from curves as in Figure S6b while the lines are simulated according to the model as previously explained (Matulis *et al.*, 2005).

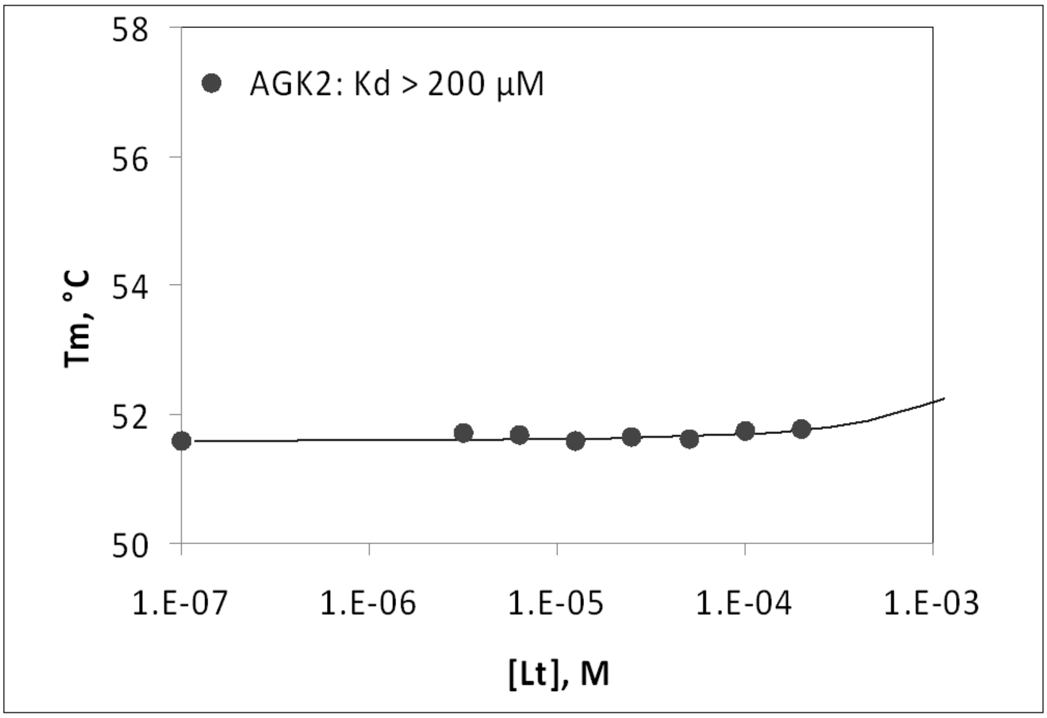

Table S1. Quantification of deacetylation reaction as observed by ^{1}H-NMR.

		Normalised Relative Integral Intensity of signal at 2.04 ppm	% deacetylation
(a)	Before adding enzyme	100	0
(b)	No inhibitor control	4	96
(c)	25 µM	11	89
(d)	50 µM	16	84
(e)	100 µM	34	66
(f)	200 µM	64	36
(g)	500 µM	89	10

Figure S10. ^{1}H and ^{13}C-NMR of Tenovin analogues.

Tenovin-36

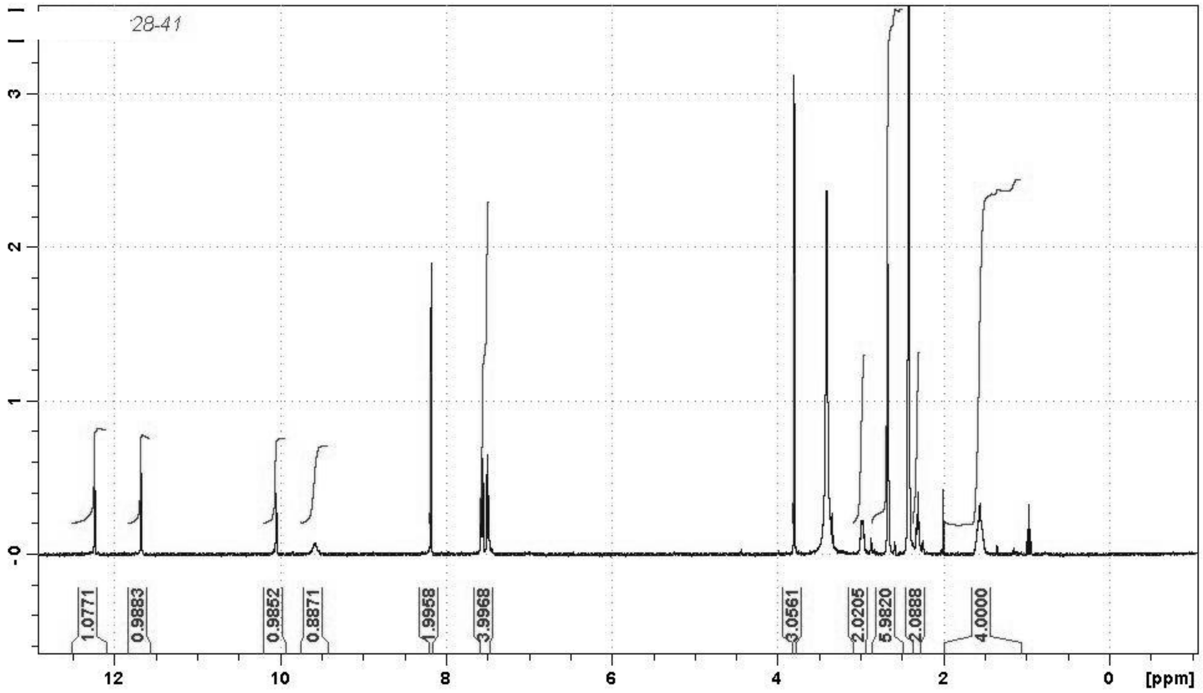

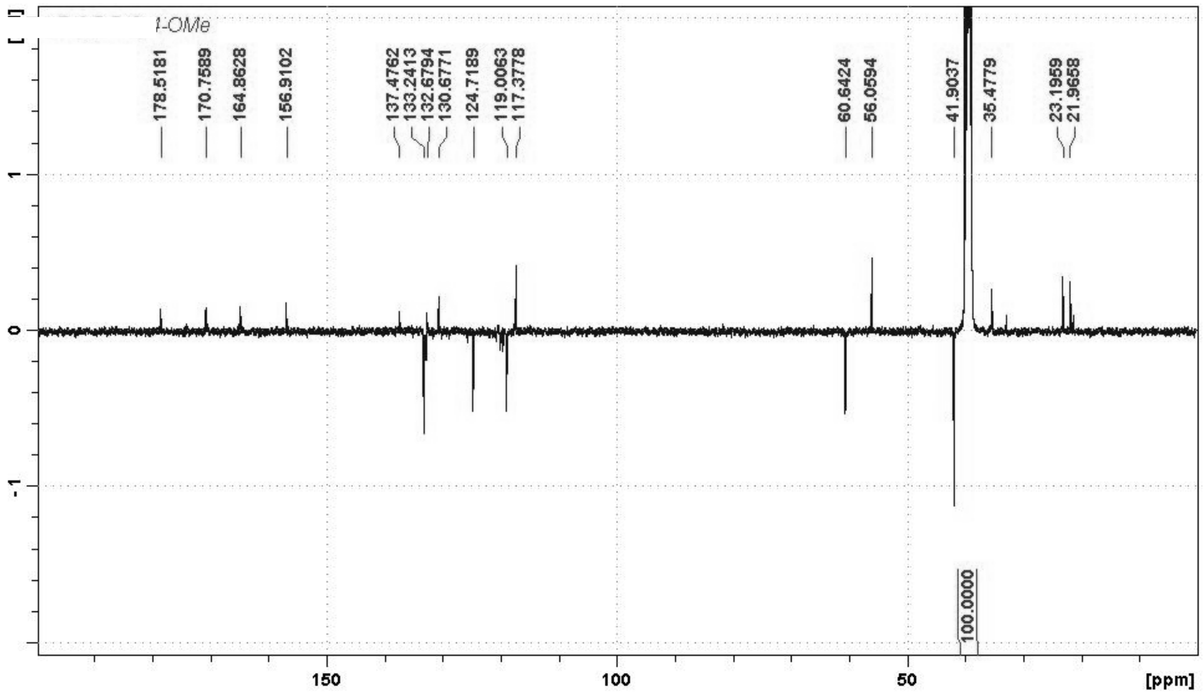

Tenovin-37

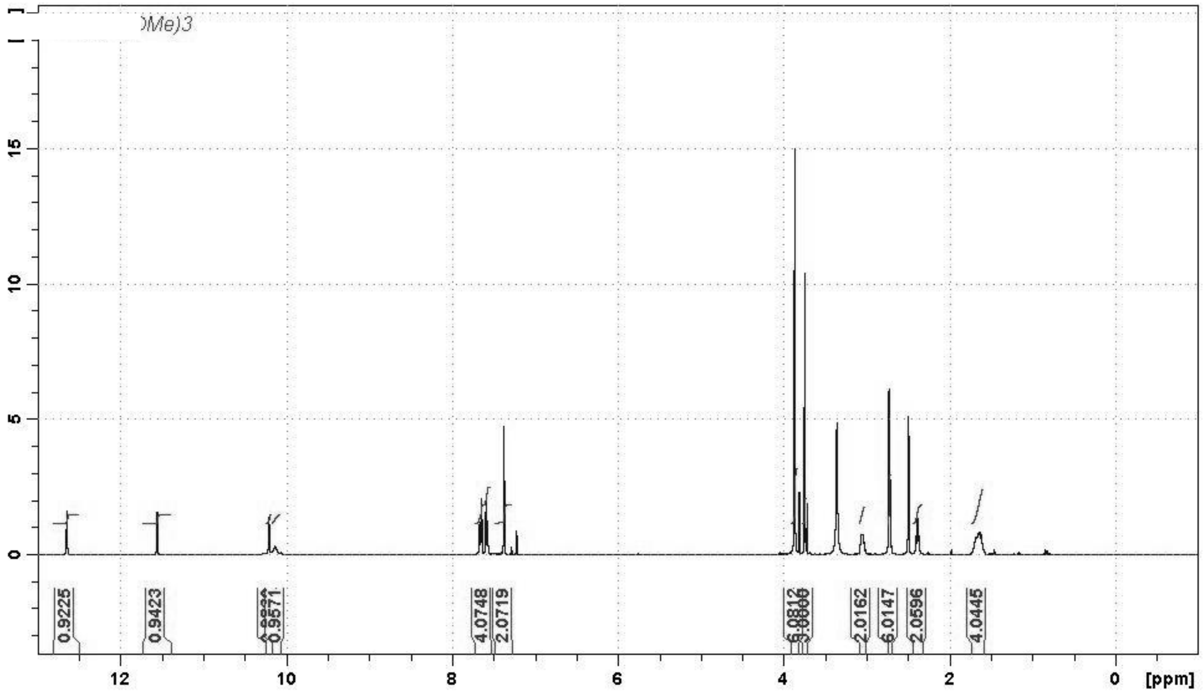
0.9225
0.9423
0.9571
4.0748
2.0719
2.0162
6.0147
2.0596
4.0445
[ppm]

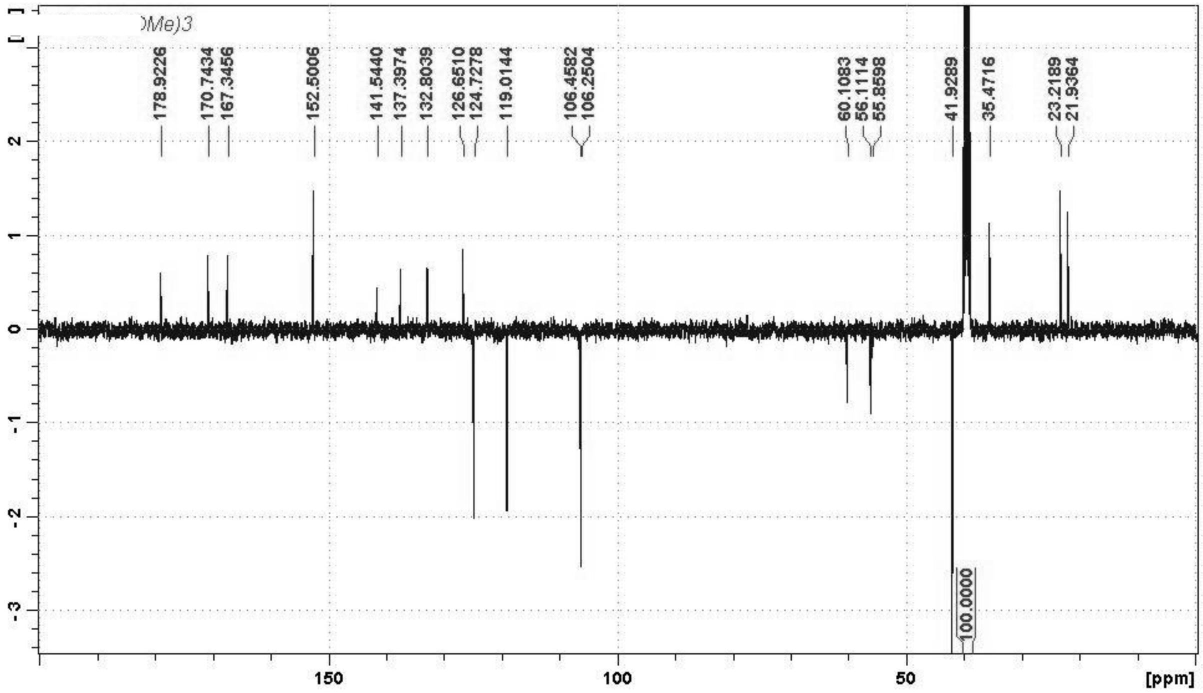
178.9226
170.7434
167.3456
152.5006
141.5440
137.3974
132.8039
126.6510
124.7278
119.0144
106.4582
106.2504
60.1083
56.1114
55.8598
41.9289
35.4716
23.2189
21.9364
100.0000
[ppm]

Tenovin-38

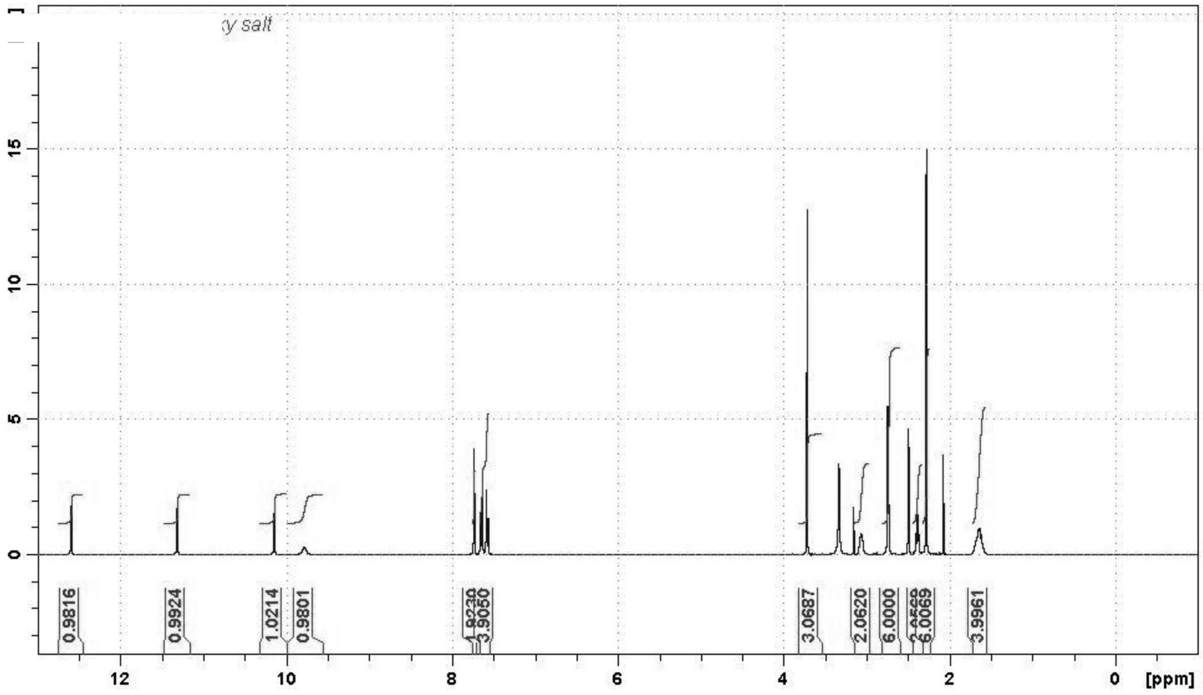

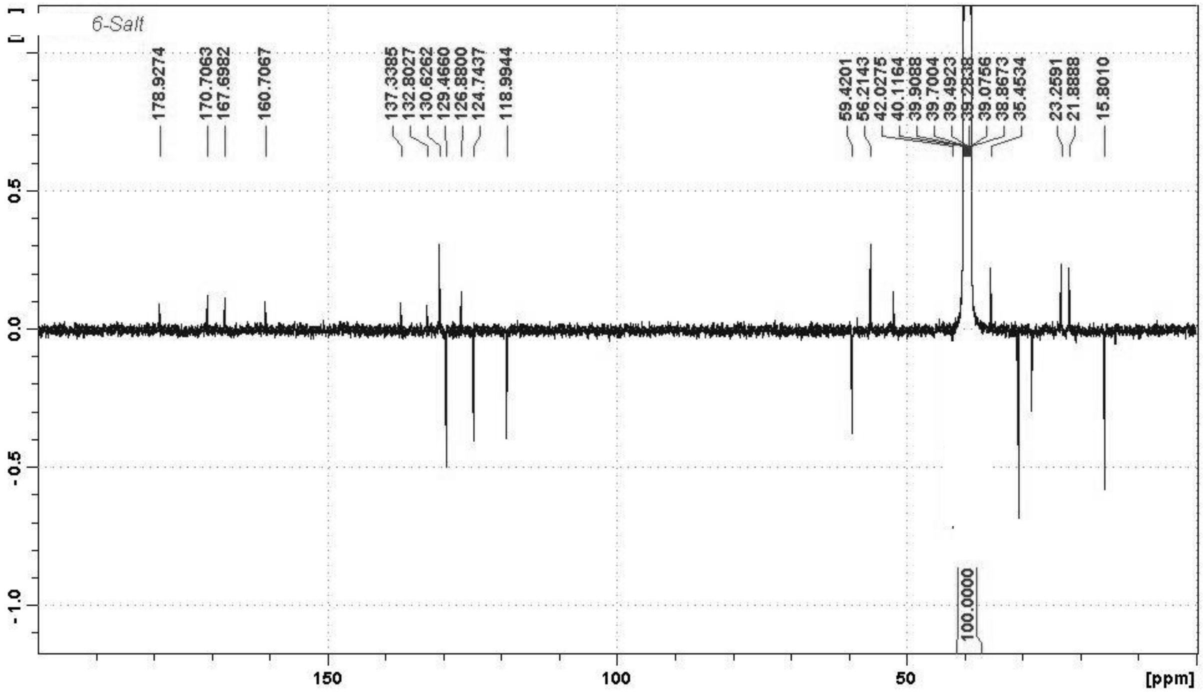

Tenovin-39

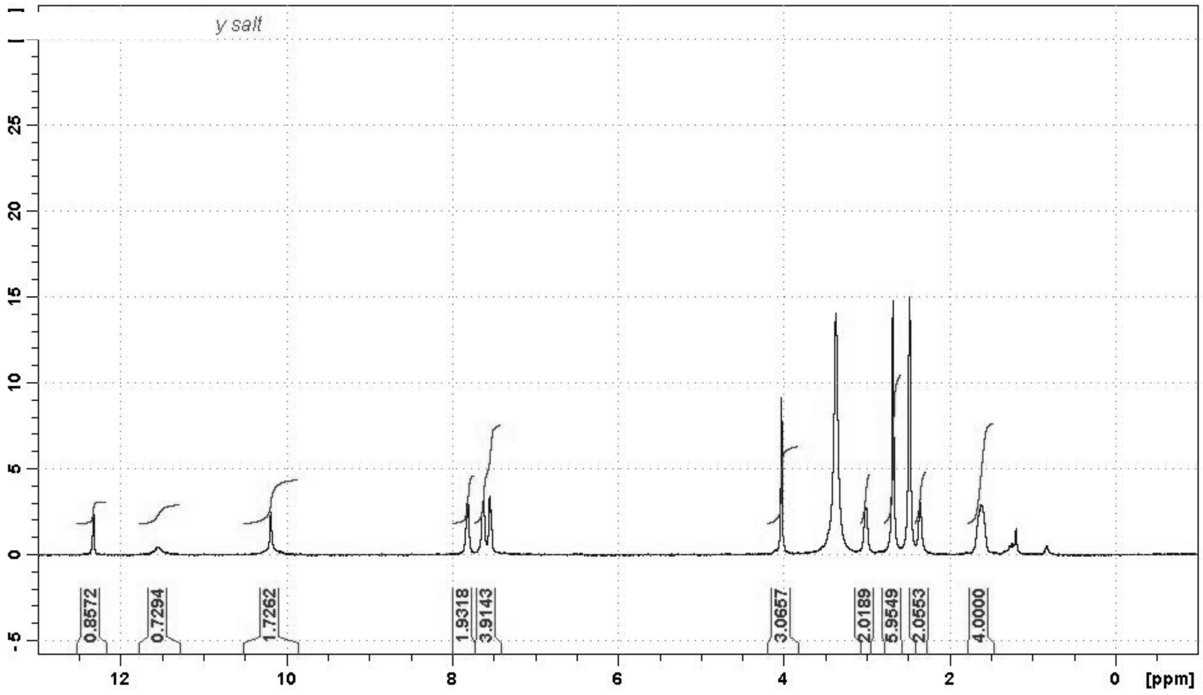

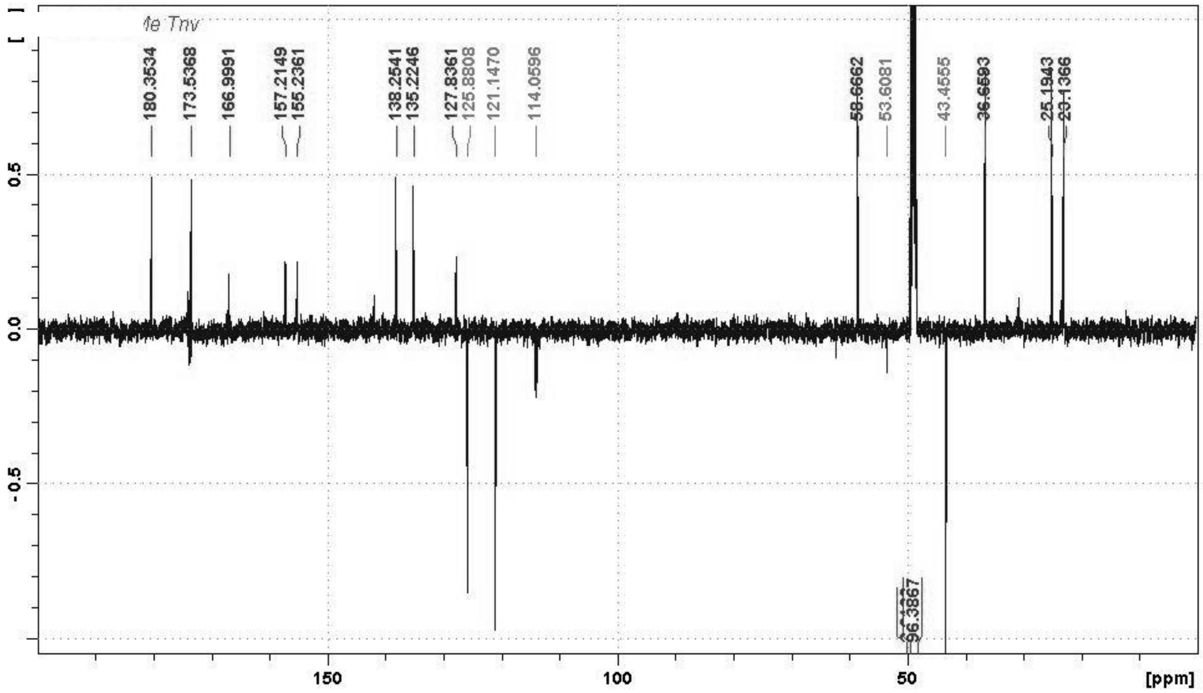

Tenovin-40

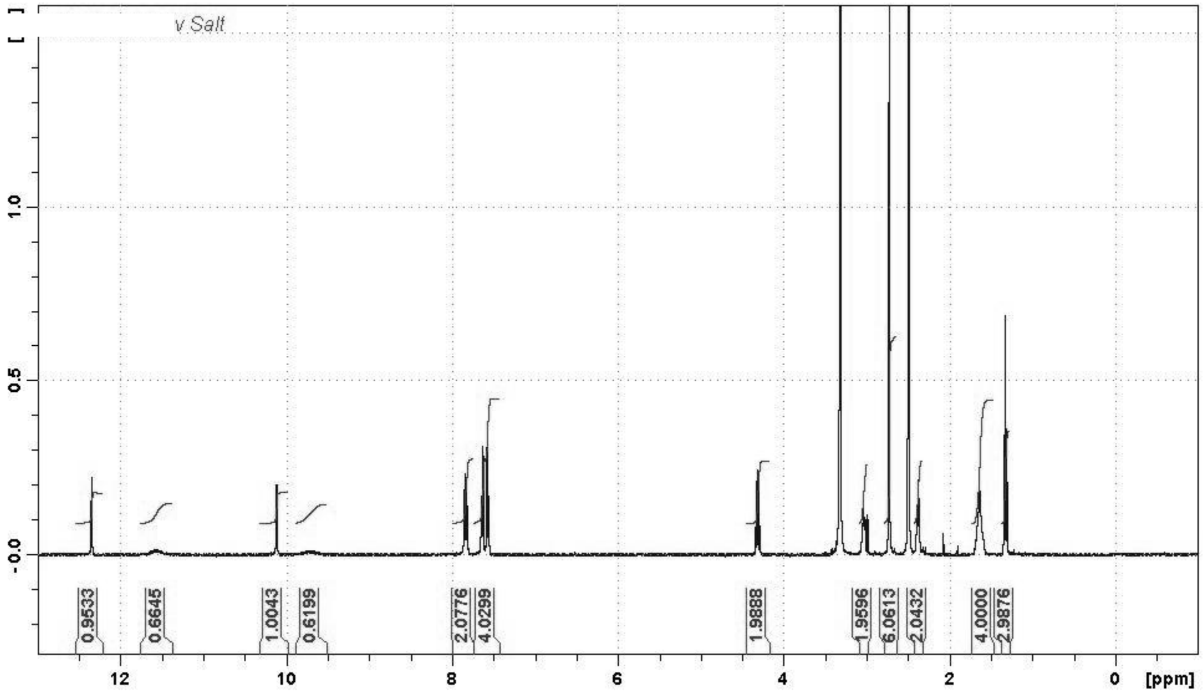
v Salt
0.9533
0.6645
1.0043
0.6199
2.0776
4.0299
1.9888
1.9596
6.0613
2.0432
4.0000
2.9876
[ppm]

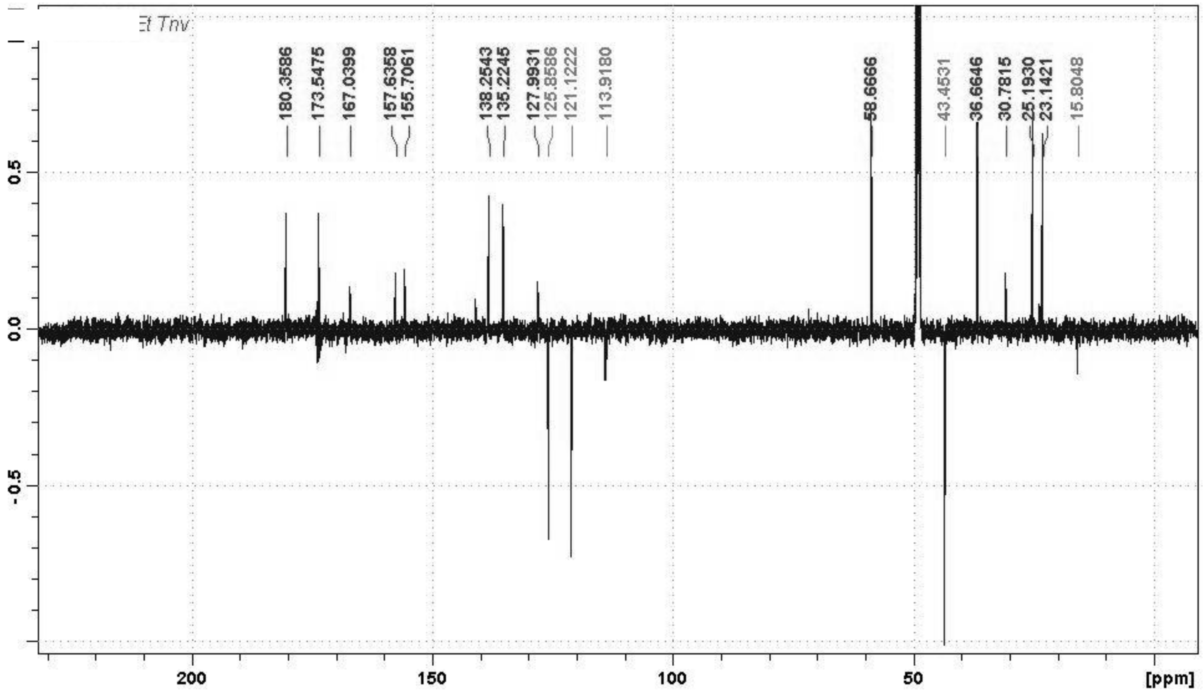
Et Tnv
180.3586
173.5475
167.0399
157.6358
155.7061
138.2543
135.2245
127.9931
125.8586
121.1222
113.9180
58.6666
43.4531
36.6646
30.7815
25.1930
23.1421
15.8048
[ppm]

Tenovin-41

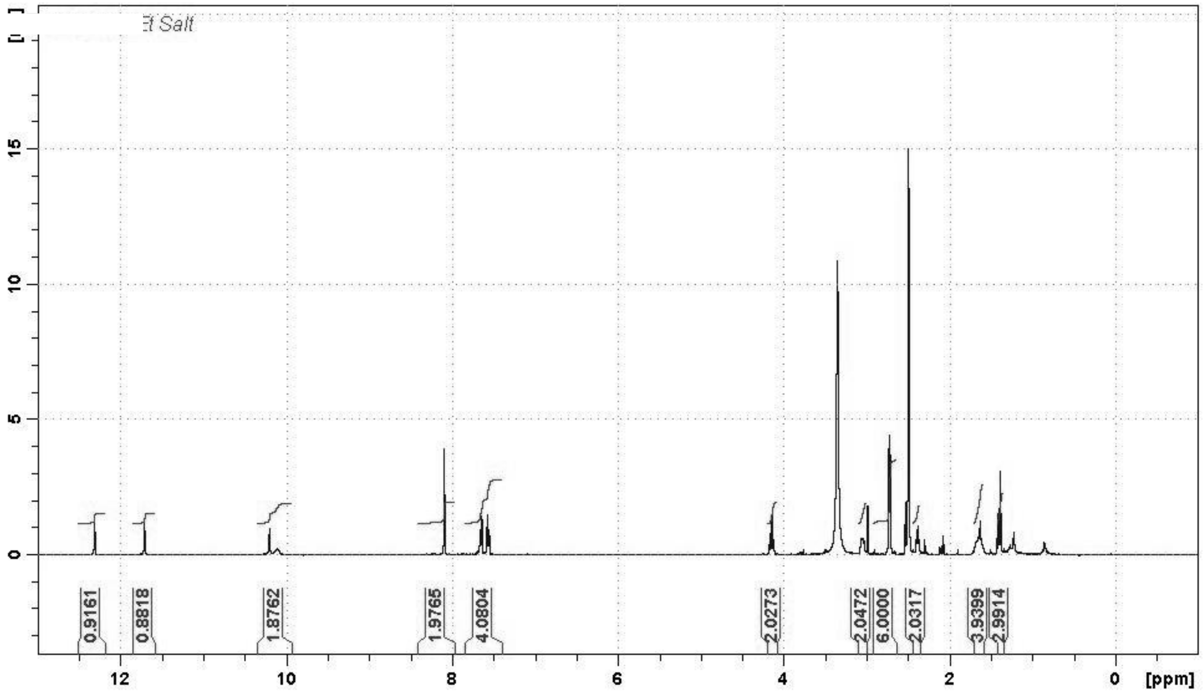
Et Salt
0.9161
0.8818
1.8762
1.9765
4.0804
2.0273
2.0472
6.0000
2.0317
3.9399
2.9914
[ppm]

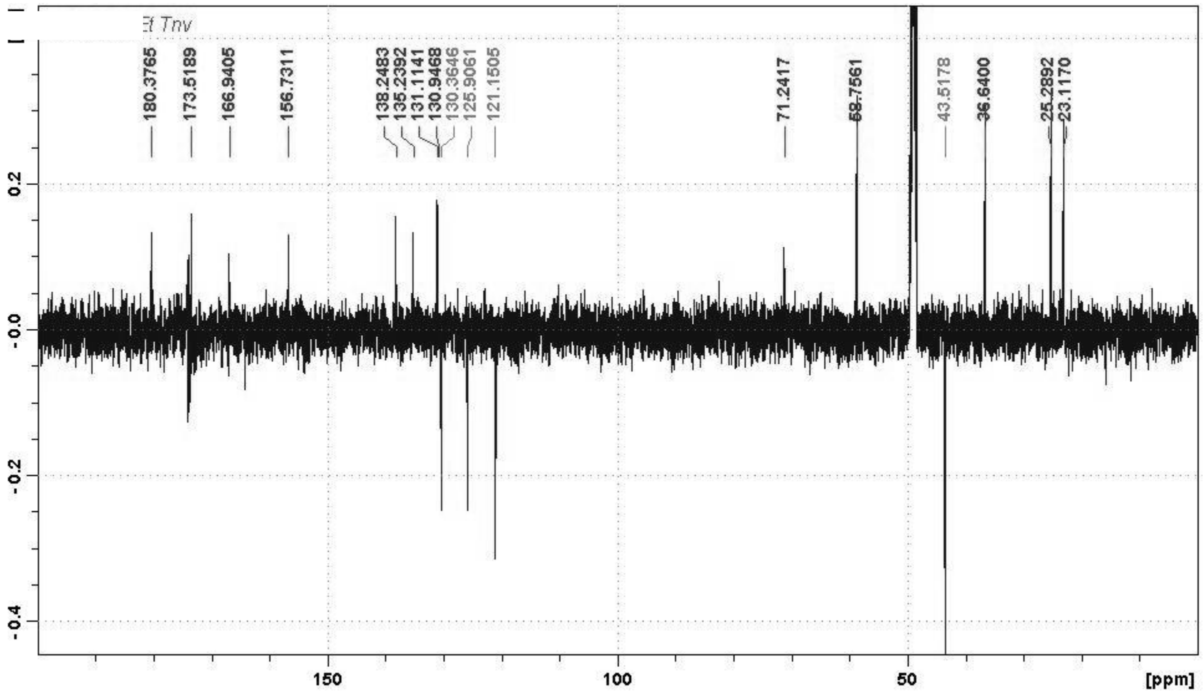
Et Tnv
180.3765
173.5189
166.9405
156.7311
138.2483
135.2392
131.1141
130.9468
130.3646
125.9061
121.1505
71.2417
58.7561
43.5178
36.6400
25.2892
23.1170
[ppm]

Tenovin-42

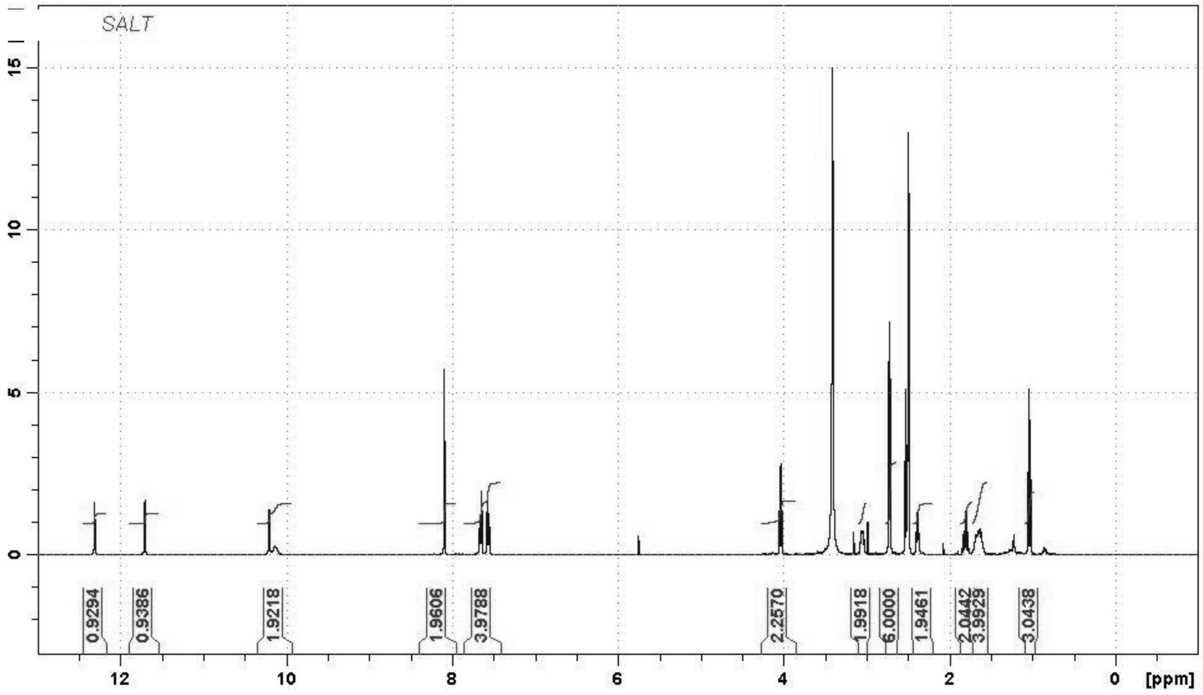
SALT
0.9294
0.9386
1.9218
1.9606
3.9788
2.2570
1.9918
6.0000
1.9461
2.0442
3.9929
3.0438
[ppm]

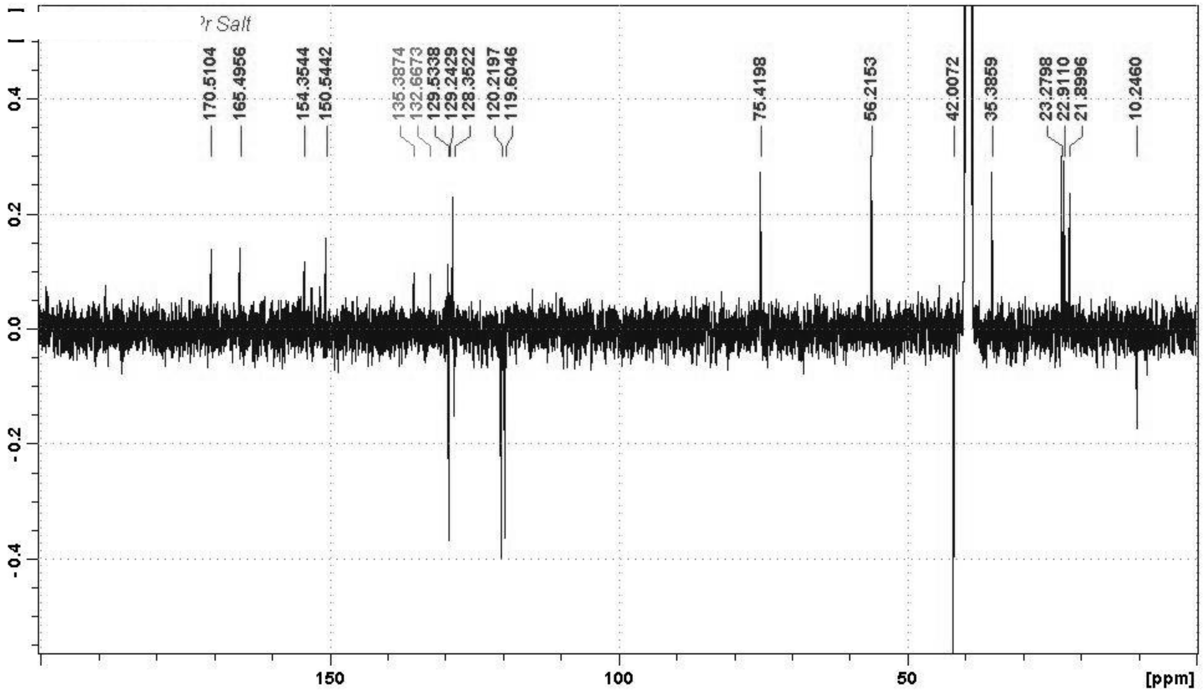
170.5104
165.4956
154.3544
150.5442
135.3874
132.6673
129.5338
129.2429
128.3522
120.2197
119.6046
75.4198
56.2153
42.0072
35.3859
23.2798
22.9110
21.8996
10.2460
[ppm]

Tenovin-43

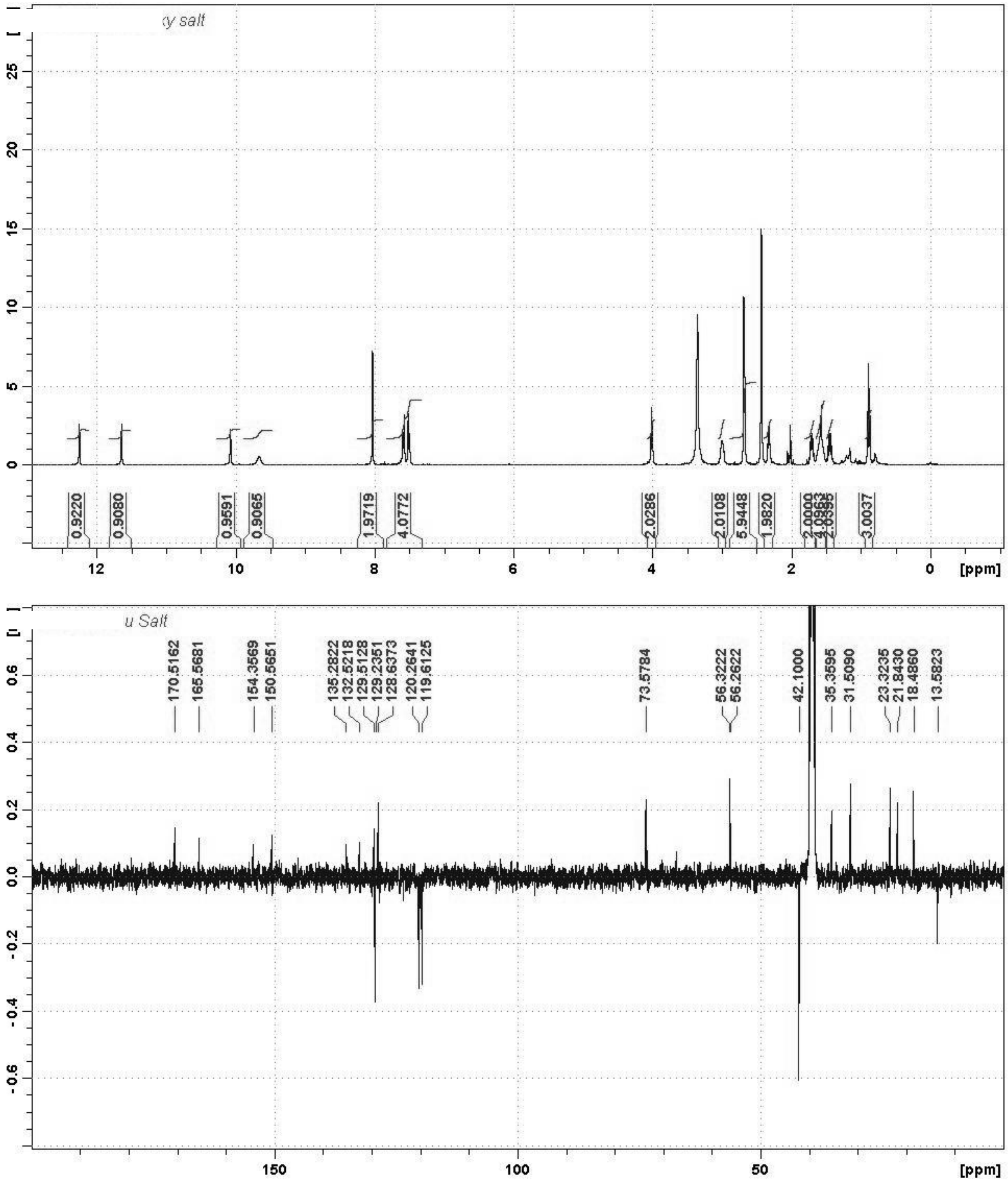

y salt
0.9220
0.9080
0.9591
0.9065
1.9719
4.0772
2.0286
2.0108
5.9448
1.9820
2.0000
4.0963
2.0395
3.0037
[ppm]
u Salt
170.5162
165.5681
154.3569
150.5651
135.2822
132.5218
129.5128
129.2351
128.6373
120.2641
119.6125
73.5784
56.3222
56.2622
42.1000
35.3595
31.5090
23.3235
21.8430
18.4860
13.5823
[ppm]

Tenovin-44

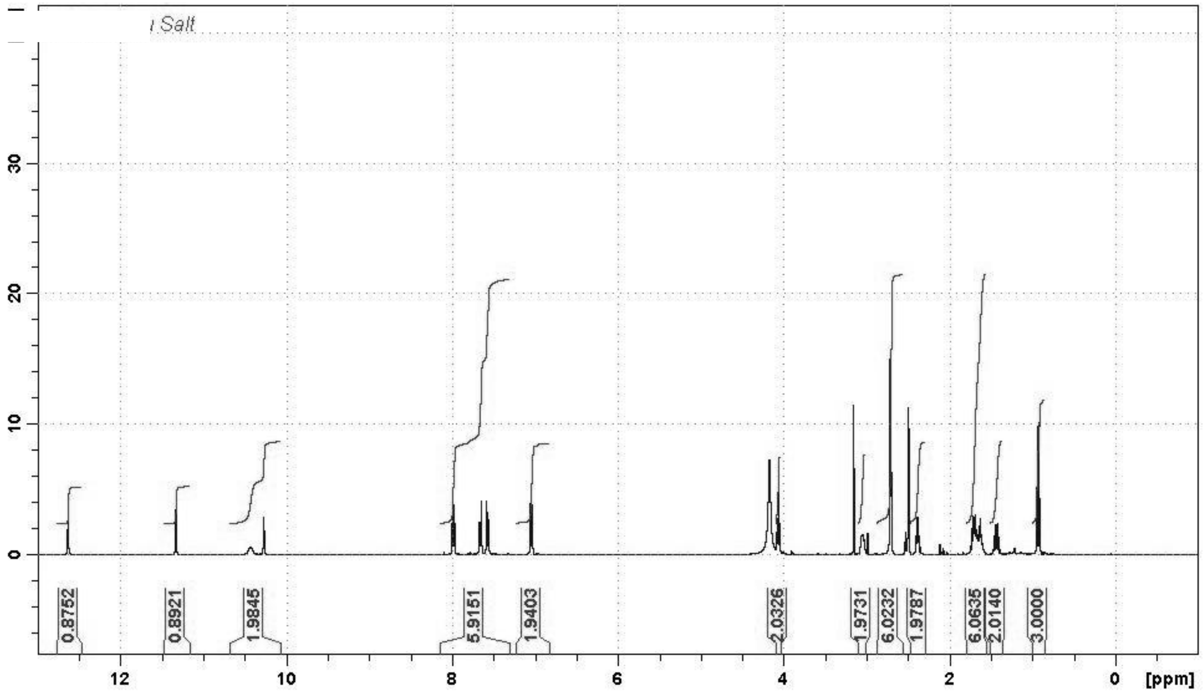

Salt
0.8752
0.8921
1.9845
5.9151
1.9403
2.0326
1.9731
6.0232
1.9787
6.0635
2.0140
3.0000
[ppm]

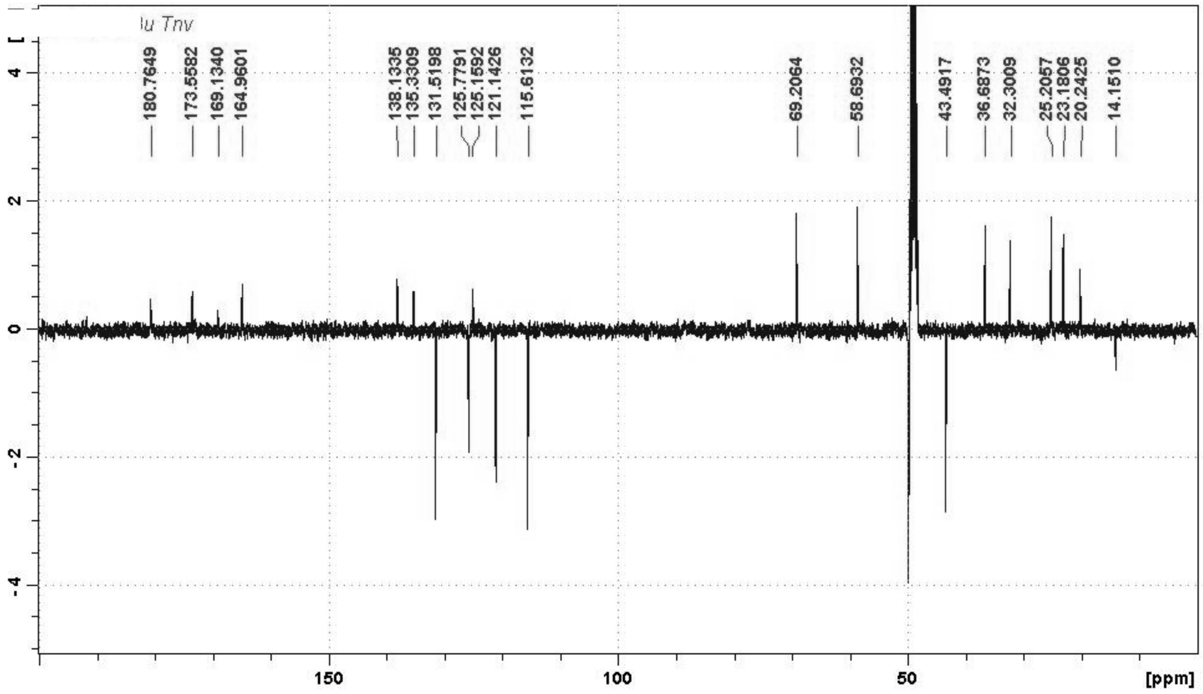

Tnv
180.7649
173.5582
169.1340
164.9601
138.1335
135.3309
131.5198
125.7791
125.1592
121.1426
115.6132
69.2064
58.6932
43.4917
36.6873
32.3009
25.2057
23.1806
20.2425
14.1510
[ppm]
